# Performance of deep learning methods for spatial gene expression prediction from histology images: a comprehensive assessment

**DOI:** 10.1093/bioadv/vbag202

**Published:** 2026-07-20

**Authors:** Jacky Xie, Xiao Tan, Yuanhao Jiang, Hao Nguyen, Nan Ye, Quan Nguyen

**Affiliations:** Institute for Molecular Bioscience, The University of Queensland, St Lucia, QLD 4072, Australia; School of Mathematics and Physics, The University of Queensland, St Lucia, QLD 4067, Australia; Institute for Molecular Bioscience, The University of Queensland, St Lucia, QLD 4072, Australia; QIMR Berghofer Medical Research Institute, Herston, QLD 4006, Australia; Institute for Molecular Bioscience, The University of Queensland, St Lucia, QLD 4072, Australia; Institute for Molecular Bioscience, The University of Queensland, St Lucia, QLD 4072, Australia; QIMR Berghofer Medical Research Institute, Herston, QLD 4006, Australia; School of Mathematics and Physics, The University of Queensland, St Lucia, QLD 4067, Australia; Institute for Molecular Bioscience, The University of Queensland, St Lucia, QLD 4072, Australia; QIMR Berghofer Medical Research Institute, Herston, QLD 4006, Australia

## Abstract

**Motivation:**

Spatial transcriptomics enables spatially-resolved measurement of gene expression in tissues, but its widespread adoption has been limited by high cost. Predicting gene expression profiles from histology images using deep learning has recently attracted broad interest as a promising and cost-effective spatial transcriptomics solution, yet the strengths and limitations of existing methods have not been comprehensively assessed.

**Results:**

We reviewed current approaches and compared seven algorithms across six datasets spanning four cancer types and a non-cancer disease, covering three spatial protocols and a comprehensive set of performance measures. Unlike previous studies that focused only on highly variable genes, we also analysed the predictability of genes functionally relevant to disease. We further assessed out-of-domain performance, the effect of data transformation techniques, the use of predicted gene expression for detecting tissue domains, and the benefit of integrating histopathology foundation models. Our results provide new quantitative insights into the limitations and promise of deep learning based spatial transcriptomics methods, and suggest challenges and future directions.

**Availability and implementation:**

The benchmarking pipeline and source code are available at https://github.com/BiomedicalMachineLearning/DeepHis2Exp.

- We performed extensive computational experiments for in silico spatial transcriptomics methods, covering seven existing methods, six cancer and non-cancer datasets, five performance metrics, and different groups of target genes for prediction.
- Several algorithms perform better on both in-domain and out-of-domain data, but none outperforms all the others across all settings. We use the top performing methods to determine a group of functionally relevant genes that can be predicted to relatively higher accuracy.
- A suitable gene expression transformation can be important to achieve good performance, but image transformations appear to have minor effects.
- Leveraging histopathological foundation models for feature extraction improves performance.
- Predicted gene values can map tissue domains that align with pathological annotations comparable to using the original spatial data.
- in silico spatial transcriptomics is promising for diagnostic applications, but performance improvements are needed before they can be reliably applied in practice.
- Our publicly available and reproducible source code provides a benchmarking pipeline useful for further research.

## 1 Introduction

Spatial transcriptomics (ST) is a rapidly developing technology, producing information on both unseen molecular signatures and imaging morphological features of tissue samples Marx (2021). As an example, one of the most common technologies is the 10× Genomics Visium platform (Ståhl *et al*. 2016), which measures the expression of over 18 000 genes on thousands of tissue spots with diameters of 55–100μm in diameter and gene expression can be mapped back to the high-resolution Hematoxylin and Eosin (H&E) stained tissue image, reflecting tissue morphology. Recently, Visium HD has further refined this process by utilizing a continuous grid of 2 μm capture squares, significantly increasing the resolution of spatial molecular profiling (de Oliveira *et al*. 2025). Similarly, Stereo-seq utilizes DNA nanoball technology to achieve sub-cellular resolution, enabling the mapping of gene expression across large tissue areas with nanometer-scale precision (Chen *et al*. 2022). Furthermore, imaging-based platforms like Xenium provide targeted, high-throughput detection of RNA transcripts at sub-cellular levels. These advancements collectively enable a more granular integration of molecular data with tissue pathology, offering deeper insights into the cellular microenvironment. Integrating gene expression and morphological features from images allows characterizing the spatial heterogeneity of cancer, providing new insights into cancer tissue biology and opening an unprecedented potential for improving cancer diagnosis and prognosis (Yu *et al*. 2022). However, the high cost of a ST experiment has limited its wider adoption.

Predicting gene expression profiles from histology images using deep learning has recently gained significant attention as a promising *in silico* solution for spatial transcriptomics. If successful, such methods would enable the generation of ST data in a cost- and time-efficient manner. Several deep learning methods have been developed to predict gene expression profiles from H&E histology images. These include methods to predict non-spatially resolved bulk RNA profiles (Schmauch *et al*. 2020, Wang *et al*. 2021), and methods that can predict spatially resolved expression profiles (He *et al*. 2020, Pang *et al*. 2021, Monjo *et al*. 2022, Zeng *et al*. 2022, Tan *et al*. 2023, Xie *et al*. 2023, Hoang *et al*. 2024). In this work, we focused on evaluating these methods, providing a comprehensive review of the algorithms and benchmarking their performance.

Although previous works have highlighted the potential of deep learning approaches for predicting spatial gene profiles, the scope of empirical evaluations performed are limited in a few important aspects. First, they do not yet provide a complete picture on the generalization performance of *in silico* ST methods, due to the use of few datasets and performance metrics, and the focus on in-domain performance on highly variable genes which are not necessarily biologically relevant. Second, while different methods often use different data preprocessing transformations, there is a lack of understanding on the effects of these transformations on model performance. Moreover, there is limited understanding on the utilities of *in silico* ST methods for downstream tasks involved in a diagnostic setting. The field of computational pathology has been revolutionized by the emergence of histopathology foundation models. Unlike traditional task-specific models, foundation models are pretrained on massive, unlabeled datasets using self-supervised learning to extract highly robust and transferable visual features. Notable examples include UNI (Chen *et al*. 2024), Prov-GigaPath (Xu *et al*. 2024), and Virchow2 (Zimmermann *et al*. 2024), which have demonstrated state-of-the-art performance across diverse diagnostic tasks. A prominent example of this new generation is Virchow2, a vision transformer trained on over 3.1 million whole slide images (WSIs) across globally diverse institutions. Virchow2 employs a modified DINOv2 training objective and incorporates mixed magnification (from 5× to 40×) to capture both high-resolution cellular details and broader architectural context. While these foundation models offer significantly richer feature representations than the standard CNN backbones typically used in morphological feature extraction in ST prediction, their integration with current gene expression prediction frameworks remains an underexplored frontier.

To address these limitations and facilitate further research, we systematically benchmarked the performance of seven existing methods, testing across different tissue types, across experimental protocols and data from different laboratories. Our experiments also cover both in-domain and out-of-domain settings, different target sets including both highly variable genes and functionally relevant genes, and performance is evaluated across five performance metrics. We also evaluated the effects of different data augmentation and preprocessing strategies, and the effectiveness of *in silico* ST for spatial domain detection. In addition, we investigated the use of existing pathology foundation models for producing embeddings to enhance predictive accuracy and generalization capabilities for the top performing models.

First, we review the architectures and algorithms of seven existing deep-learning models developed for predicting spatial gene expression profiles from histology images. Next, we outline our benchmarking methodology and experimental design for assessing these seven models. We then present and analyse the benchmarking results, explaining the observed differences in performance based on the different choices of model architectures and datasets.

Finally, we discuss the limitations of this benchmarking study and highlight the challenges associated with applying deep learning models to predict spatial transcriptomic profiles.

## 2 Review of deep learning methods

We identify seven existing deep learning methods used for predicting ST gene profiles, summarised in Table 1. We provide a brief overview of the main ideas and similarities between each method; a more detailed summary of each method is provided in the Supplementary Materials.

**Table 1:** Summary of published algorithms.

No.	Algorithm	Year	Model	Sample	Technology	Baselines
1	ST-Net (He *et al.* 2020)	2020	CNN	Breast cancer	Legacy	
2	HisToGene (Pang *et al.* 2021)	2021	CNN, Transformer	Breast cancer, skin	Legacy	ST-net
3	Hist2ST (Zeng *et al.* 2022)	2022	CNN, Transformer, GNN	Breast cancer, skin	Legacy	ST-net, HisToGene
4	DeepSpaCE (Monjo *et al.* 2022)	2022	CNN	Breast cancer	Visium	ST-net
5	BLEEP (Xie *et al.* 2023)	2023	CLIP	Liver tissue	Visium	ST-net, HisToGene
6	STimage (Tan *et al*. 2023)	2023	CNN	Breast cancer	Visium, legacy	ST-net, HisToGene, Hist2ST
7	DeepPT (Hoang *et al.* 2024)	2023	CNN, Autoencoder	TCGA data	–	

‘Baselines’ column indicates which algorithms were compared against in the original work.

Most methods (ST-Net, HisToGene, Hist2ST, STimage, DeepSpaCE, DeepPT) share the same high-level encoder-predictor structure: an image encoder is used to extract features from image patches, then a prediction head is used to predict the gene expression profiles using the extracted features. The encoder can be sample-level or patch-level encoders: a sample-level encoder (e.g. the transformer used in HisToGene and Hist2ST) takes image patches at other locations into account when encoding the image patch at a single location, while a patch-level encoder (e.g. the DenseNet-121 used in ST-Net) encodes each image patch separately. Some methods such as STimage and Hist2ST additionally include a distribution module to predict the distribution of gene expression values rather than a point value. The aforementioned methods are trained in a multivariate regressive way; the exception is BLEEP, which infers the gene expression by querying through nearest-neighbors in a learned joint embedding space for image and gene expression. Moreover, these methods employ different techniques to preprocess image and gene expression data, as shown in Table S1.

## 3 Benchmarking methodology

**Figure 1:**
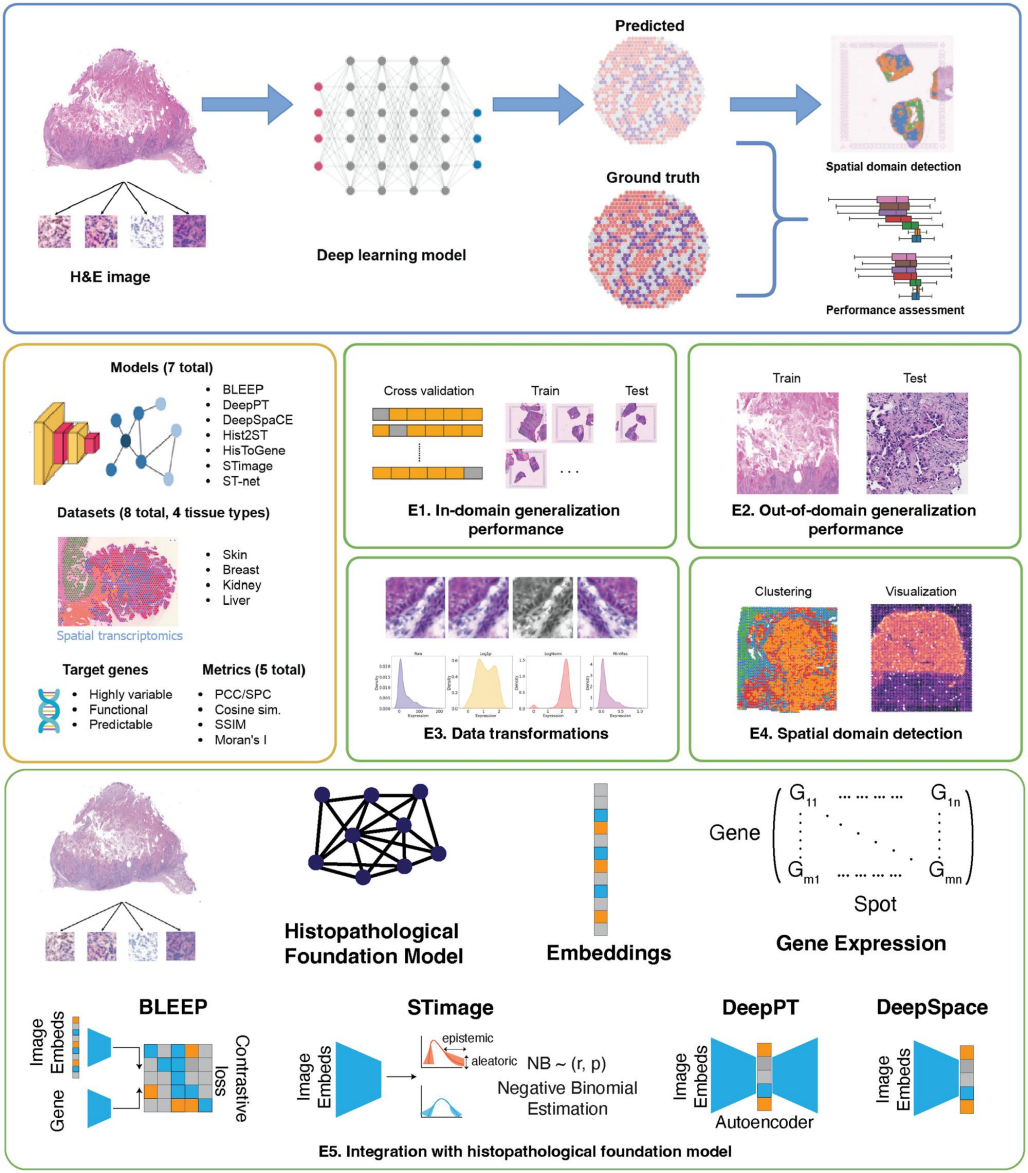
Schematic of current deep learning methods and scope of review. The top diagram outlines the gene expression prediction problem for spatial transcriptomics and the workflow shared by the deep learning model methods. The predicted gene expression can be compared to the ground-truth using a variety of metrics and the predicted values can be used for spatial domain detection. The scope and features of this benchmarking work are displayed at the bottom, with a summary of the models and datasets considered and overview of the benchmarking comparisons and experiments (E1–E5).

Figure 1 illustrates the general workflow for evaluating *in silico* ST methods that are based on deep learning models. Methods were run following the default settings as reported in the original papers and our benchmarking code is publicly available on GitHub. More details about the datasets, data preprocessing and augmentation are described in the Supplementary Materials. Figure 1 also highlights the main features of this benchmarking study. We explain the choice of the datasets, the choice of the target genes, and the performance metrics below. Using this set-up, we conducted a number of experiments grouped into five categories that assess the performance of each method in in-domain (E1) and out-of-domain (E2) settings. We also use a reference model to assess different data processing and augmentation strategies (E3) and also compare the quality of results when model predictions are used for spatial domain detection (E4), and we evaluate integration with histopathological foundation models (E5).

### 3.1 Datasets

We used multiple spatial transcriptomic datasets consisting of various cancer/disease types generated by the Visium technology and the legacy ST technology at different resolutions. The datasets are summarized in Table S3 and detailed further in the Supplementary Materials. To increase the sample size within the breast cancer data, we combined two 10X BC Visium datasets and the Wu *et al*. BC Visium dataset, hereafter named the BC Visium dataset. After this reorganization, there are a total of six datasets available for benchmarking work. Both biological variation (e.g. patient, cancer type) and technical variation (e.g. ST protocol, laboratory) are present, which allows us to assess how models perform across datasets with different biological and technical characteristics and variation observed in real application settings.

### 3.2 Target genes

Predicting the whole transcriptome is highly challenging, and current methods have focused on a subset of most abundant or most variable genes. In our benchmarking experiments, we considered two sets of target genes: (i) highly variable genes, and (ii) functionally relevant genes.

Highly variable genes (HVGs) have been used in most previous works as target genes (Zeng *et al*. 2022). For Her2ST and cSCC, we reused those 785 and 134 HVGs respectively as chosen in Zeng *et al*. (2022). For the other datasets, we selected the top 1000 highly variable genes as follows: first we filtered out genes that were expressed at less than 10% of the spots in any sample, then applied normalization and log1p transformation to the gene expression values for each sample, and finally selected the 1000 genes with the highest variance for their expression values in all samples. Normalization was applied to account for technical variation between the spots in a sample, and log1p transformation is applied to squash the range of the gene expression values from 0 to $10^{6}$ to 0 to 6, so that the variance is not dominated by the large expression values.

Differing from previous studies that benchmarked only highly variable genes, in this work we used a new set of functionally relevant genes (FGs) to assess model performance. We identified common predictable genes within this set. As only a small proportion of HVG genes are functionally related to cancer or cell type identities, we posited that it is necessary to assess the performance based on a large, consensus set of functionally relevant genes. Details for the set of functionally relevant genes are provided in the Supplementary materials.

Our study further reports the methods’ performance on the most predictable FGs across the various datasets, obtained by taking the intersection of the top 500 predictable genes ranked by the median Pearson correlation for the top four best performing methods within the dataset.

### 3.3 Performance metrics

We evaluated the gene expression predictions using five metrics: Pearson correlation coefficient (PCC), Spearman rank correlation coefficient (SPC), cosine similarity (CSIM), the structural similarity index (SSIM), and we introduced a new spatial correlation measure between the predictions and the observations, inspired by Moran’s *I* to measure how well the spatial autocorrelation in the observed values can be captured in the prediction. We shall use Moran’s *I* to refer to this correlation measure. A detailed description and explanation of the choice for each method is provided in the Supplementary materials.

To formally compare the seven methods, we performed a rank-based non-parametric analysis on the predictable functionally relevant gene set. For each Dataset/setting × metric block, we first summarized the gene/slide-level scores for each method using the median. Cosine similarity was excluded from this statistical comparison because it was less discriminative in our benchmark and does not involve normalization/standardization in the same way as the correlation-based and spatial metrics. The remaining four metrics were Pearson correlation, Spearman correlation, SSIM, and Moran’s *I*. Methods were ranked within each block, with rank 1 assigned to the highest median score. We then applied the Friedman test to assess overall differences among methods, followed by Nemenyi *post-hoc* comparisons and a critical-difference diagram to visualize differences in average ranks.

### 3.4 Benchmarking foundation model encodings

To assess the potential of domain specific representation learning, we investigated the impact of replacing generic ImageNet pretrained encoders or CNNs with Virchow2, a state-of-the-art computational pathology foundation model. The hypothesis underpinning this experimental design is that a visual encoder exposed to millions of diverse histopathology images during pretraining will yield a feature space that is not only more distinctive but also more biologically and semantically aligned with transcriptomic profiles. This alignment is expected to reduce the complexity of the mapping function required by downstream regressors, thereby improving generalization, particularly in OOD settings where stain and scanner variations typically degrade the performance of CNN-based models. To benchmark the efficacy of Virchow2, we restructured the image encoding modules of four state-of-the-art methods: DeepPT, DeepSpaCE, BLEEP, and STimage. The original image feature extractor backbones were removed, and the architectures were modified to accept the pre-computed 2560-dimensional Virchow2 embeddings. Further details for this experiment, including dataset and gene set choice, are provided in the Supplementary Materials.

## 4 Results

### 4.1 ID generalization performance

We evaluated the methods in the in-domain setting using leave-one-out cross-validation (LOOCV) for each dataset. Specifically, we held out one sample, trained the model on the remaining samples, and then performed prediction on the hold-out sample to compute the five-performance metrics. We used both the functionally relevant gene set and the highly variable gene set to evaluate the ID performance.

**Figure 2:**
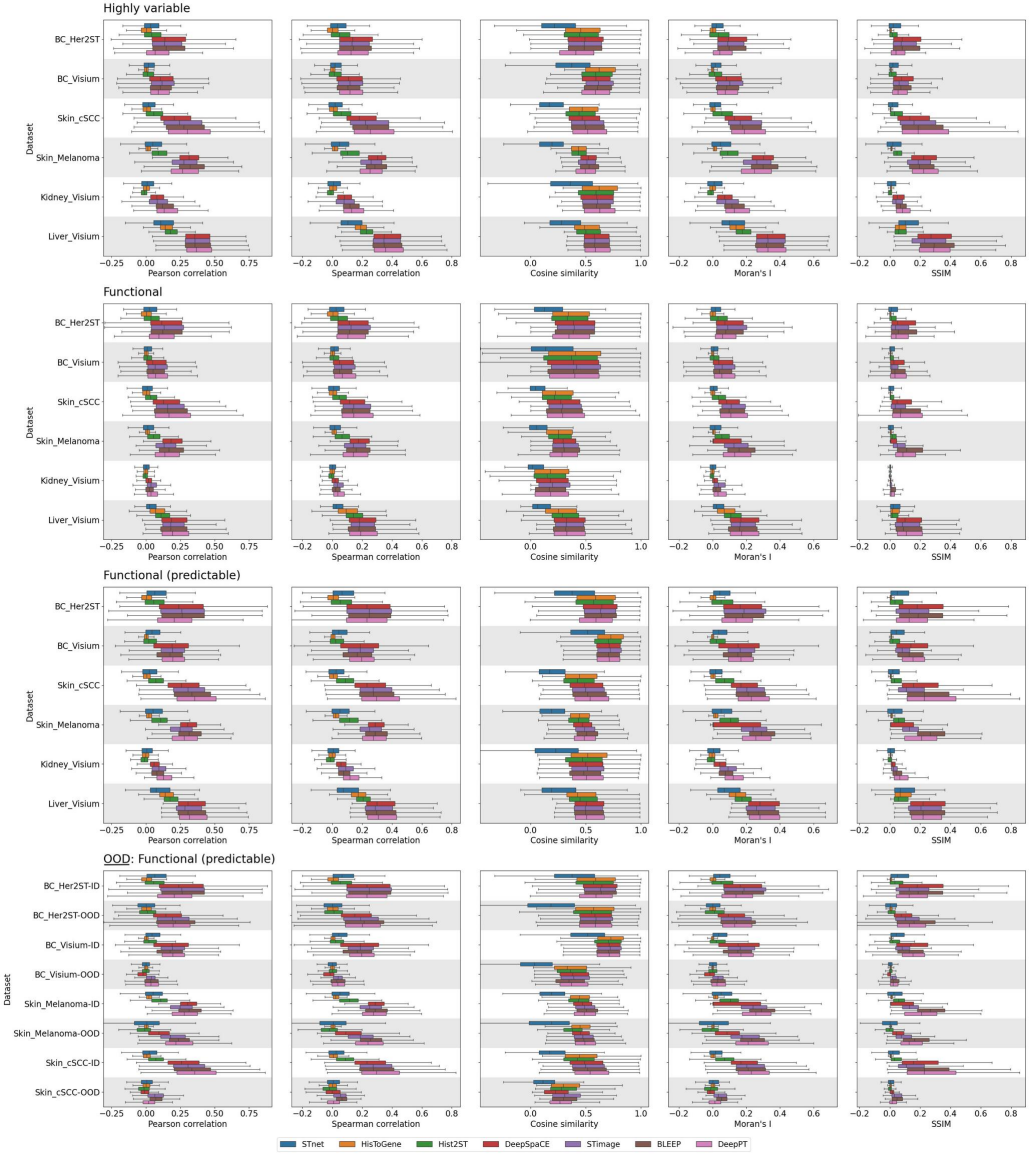
Performance of predicting genes in the in-domain (ID) and out-of-domain (OOD) settings. Each performance metric is calculated at the gene-wise level (i.e. for each gene, across all spots) and we display the box plots for the scores of all the genes in the corresponding gene sets. The plots in the top three rows show results for the in-domain (ID) experiments with different target gene sets (i.e. top 1000 variable genes, 1630 functionally relevant genes, and top 500 most predictable functionally relevant genes). We evaluated each model on the ID setting using leave-one-out cross-validation. The plots in the last row correspond to the out-of-domain (OOD) generalizability experiment, with results showing the performance for predictable functionally relevant genes. Here, the OOD dataset labels refer to the following model training setup—BC_Her2ST-OOD: trained on skin cancer, cSCC, tested on Her2ST. BC_Visium-OOD: trained on melanoma, tested on breast cancer Visium. Skin_Melanoma-OOD: trained on cSCC, tested on melanoma. Skin_cSCC-OOD: trained on melanoma dataset, tested on cSCC.

Overall, our results indicate that across different metrics and datasets, four models, namely DeepSpaCE, STimage, BLEEP, and DeepPT, generally outperformed Hist2ST, HisToGene and ST-Net for both the highly variable and functionally relevant genes (Figure 2). The ranking was particularly consistent across Pearson and Spearman correlation metrics, as well as the more specialized metrics SSIM and Moran’s *I*, while much less so for the cosine similarity. In fact, the cosine similarity is the only metric which does not involve normalization/standardization and tends to produce much higher scores than the other four metrics, suggesting that it may not be a very informative metric for differentiating gene expression prediction algorithms, despite its popularity in many domains. It is also worth noting that the ranking of the models was similar across different spatial resolutions (100 µm versus 55 µm), different tissue types (frozen versus FFPE), disease types (different cancer types and non-cancer liver disease), and different technical variations in the number of spots and genes.

Using the benchmarking results from the full functionally relevant gene list, we identified commonly predictable genes by taking the intersection of the top 500 genes ranked by median PCC from the four best performing methods (i.e. STimage, BLEEP, DeepSpaCE and DeepPT). Table S3 displays the number of predictable genes found in each dataset. We then visualized model performance on these gene sets in Figure 2 (third row). The top 500 predictable genes (Figure 2, the third row) had a significant increase in performance across datasets and models compared to the overall predictions (top two rows). These dataset-specific most predictable gene sets were then compared across different datasets of the same organ or similar disease types. We found that a total of 66 genes were highly predictable across all datasets, 229 genes were shared across the breast cancer datasets (61% of the Her2ST genes, 67% of the BC Visium genes), and 248 genes were shared across the melanoma (68%) and skin cancer dataset (59%). Overall, we found that a large proportion of functionally relevant genes could be predicted across breast cancer and skin cancer datasets.

We further performed gene ontology analysis on the predictable functionally relevant genes for multiple disease types, as shown in Figure S1. The results suggest that the predictable functionally relevant genes were mostly associated with the immune system, cytokine signaling pathways, and signal transduction, indicating that these predictable genes can provide important information about onco-immunology interactions and disease pathological processes.

### 4.2 OOD generalization performance

In the OOD setting, we evaluated each model’s generalization ability within a single cancer type and also between cancer types. We used the functionally relevant genes for the following experiments. First, we evaluated the models’ generalization across different cancer types by training the models on one type and predicting on another type. This OOD design included four experiments: (i) the models were trained melanoma dataset and using them to infer gene expression in the skin cancer cSCC dataset (skin_cSCC-OOD); (ii) trained on cSCC and tested on melanoma datasets (Skin_melanoma-OOD); (iii) trained on skin cancer, cSCC, tested on Her2ST (BC_Her2ST-OOD); and (iv) trained on melanoma, tested on breast cancer Visium (BC_Visium-OOD; tested on the combined 10× and Wu *et al*. datasets), (Figure 2, bottom row). Interestingly, models trained on cSCC data had only a slight reduction in performance when applied to the breast cancer legacy Her2ST dataset or to the melanoma Visium dataset. The reduction was larger when applying these models to the breast cancer Visium dataset. We noted that the ranking of model performance was consistent with the results when the models were assessed on ID datasets and the top performing models, STimage, BLEEP, DeepSpaCE, and DeepPT, still produced relatively high accuracy in the OOD setting (PCC > 0.1). This suggests that OOD data may be useful for training a better model when included in the training set, which has both OOD and ID distributions.

**Figure 3:**
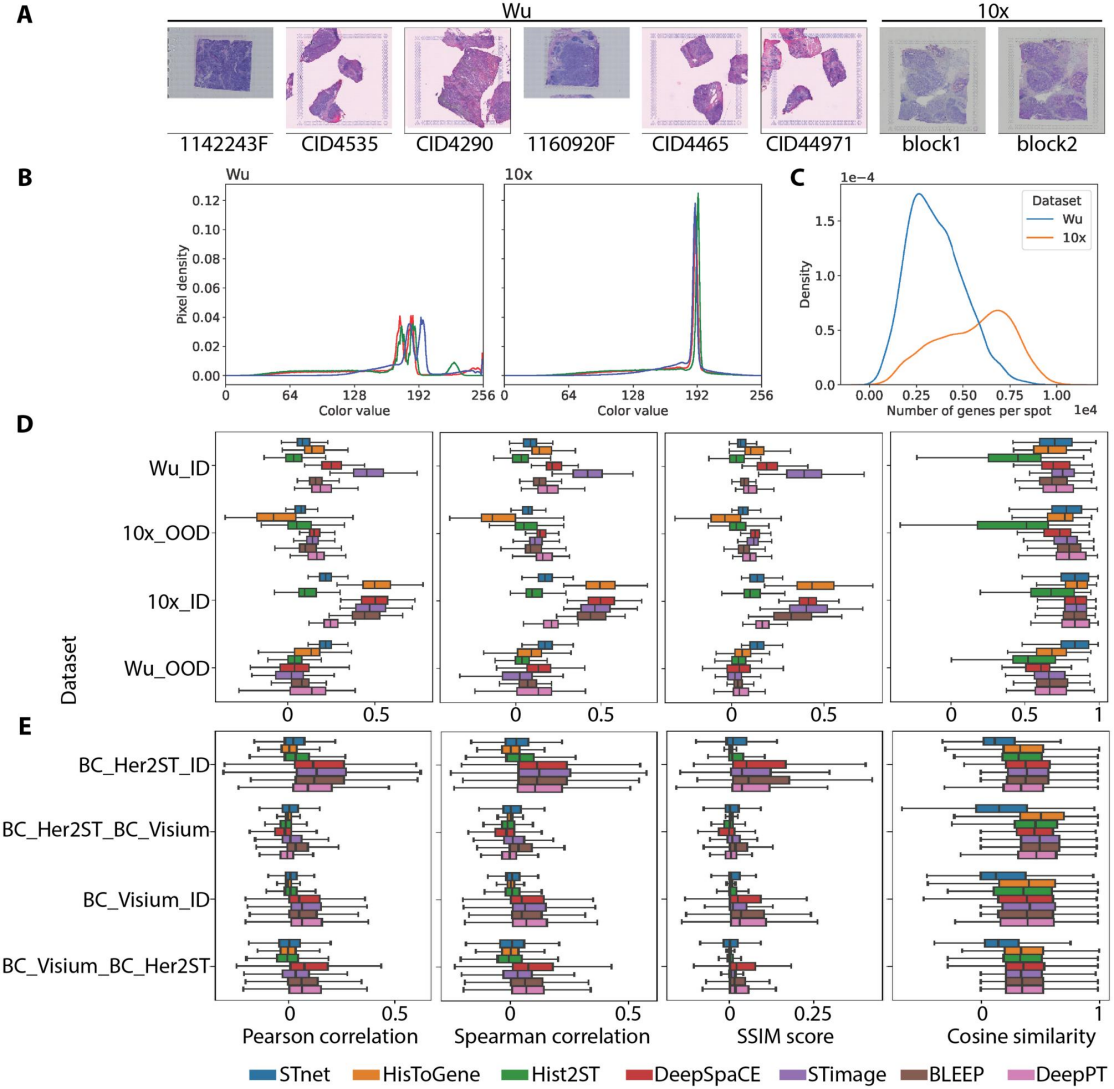
ID and OOD generalization performance within the breast cancer datasets with technical variation present. (A) Design and sample images in the Visium dataset and the dataset splits for assessing the ID and OOD generalization. (B) Comparison of the color distribution of images in the training and test datasets. We visualize the distribution of the pixel color space between the Wu *et al*. BC dataset (left) and the 10× BC dataset (right). (C) Comparison of the gene expression distribution, displaying the density of the genes detected per spot. (D) Assessment of model generalization on the in-domain breast cancer dataset with the same technology, disease type and image resolution. The first two groups (top two rows in the plot) are the results of training on the Wu *et al*. BC dataset and testing on held-out validation subset (ID) and also on the 10× BC dataset (OOD). The remaining two groups (bottom two rows) represent the results of training on the 10× BC dataset and testing on held-out validation subset (ID) and the Wu *et al*. BC dataset (OOD). The target genes used in these two experiments are the functionally relevant gene set. (E) Assessment of model generalization on in-domain for breast cancer, but using technologies that produce images of different resolution. BC_Her2ST-ID represents the result on the Her2ST dataset under the LOOCV training strategy. BC_Her2ST_BC_visium represents the result tested on the combined BC Visium dataset (Wu *et al*. and 10× Genomics), where models were trained on the Her2ST dataset. BC_visium_BC_Her2ST represents the result tested on the Her2ST dataset, where models were trained on the combined BC Visium dataset.

Next, we assessed technical variation in data within the same cancer type by comparing breast cancer samples that were collected at different geographical locations and by different laboratories, which resulted in different image quality (e.g. in stain color space) and gene distribution (Figure 3A and B). By training models on the Wu *et al*. BC dataset (55 µm) and testing on either the leave-one-out samples in the Wu *et al*. dataset (ID-Wu_etal) or the 10× breast cancer dataset (OOD-10×), we found that the performance was reduced in OOD. This result was consistent when the 10× dataset was used as the ID and the Wu *et al* dataset was used as the OOD (Figure 3D). The results demonstrate that the models did not generalize well even for the data from the same cancer type and the same spatial technology, but only different in sample cohorts and laboratories that generated the data (Figure 3D). This comparison suggests that current models are not robust to technical variation, especially when trained on data with small sample sizes.

We then investigated the effects of sample size and image resolution on the model performance. We compared performance in the ID and OOD breast cancer setting where the spatial resolutions varied. We trained the models on the Her2ST (36 samples) and tested them on the higher resolution BC Visium dataset (9 samples) (Figure 3E). We then swapped the training and test datasets. It should be noted that even though the number of samples in the Her2ST dataset is larger than in the BC Visium dataset, the total number of spots within the Her2ST dataset is fewer than in the BC Visium dataset, because the higher-resolution Visium data contain more spots per sample. We found that the performance reduced markedly between ID and OOD, when training the model using the legacy ST [low resolution Her2ST dataset, with spatial resolution of (100 µm)], (Figure 3E, top two rows). The reduction was less when the model was trained using the high spatial resolution Visium dataset and was tested on the data with the same resolution (leave-one-out, ID) and for the data with a lower resolution (OOD), (Figure 3E, bottom two rows). This suggests that the model performance may be more limited by the spatial resolution than by the sample size.

We also did the Friedman test for all seven models. The Friedman test showed a significant difference among the seven methods across 32 Dataset/setting × metric blocks ($\chi^{2}=137.49$, $p=3.39\times 10^{-27}$). The average-rank analysis identified BLEEP, DeepPT, and STimage as the leading methods, with average ranks of 1.66, 2.25, and 2.62, respectively. The Nemenyi post-hoc test showed that these leading methods were not significantly different from one another at α=0.05. DeepSpaCE ranked next (average rank =4.22), while Hist2ST, ST-Net, and HisToGene had poorer, higher numerical average ranks (5.66, 5.38, and 6.22, respectively) (Figure S4).

### 4.3 Data transformations

Transformations are often applied to the H&E images and the gene expression values to improve a model’s generalization ability and its robustness to data variations arising from technology differences.

Table S1 summarizes the data transformations used in the algorithms that we evaluated. We benchmarked the effectiveness of different image and gene expression transformations using the high-performing model DeepPT and the cSCC dataset.

For image transformations, we evaluated three transformations: random grayscale, Gaussian blur, and random rotation.

Table S2 shows that all these image transformations did not appear to have a significant effect on DeepPT’s performance, despite achieving slightly better performance in some cases.

For gene expression transformations, we evaluated four transformations: log-transformation, normalized counts (log counts per million), log-transformation on normalized counts, and MinMax scaling (counts scaled to the range [0,1]). The log transformations refer to log1p.

Table S2 shows that using the log transformation alone consistently led to the best performance across all four-performance metrics. On the other hand, minmax scaling produced slightly worse performance than not applying any preprocessing. Combining the log transformation and normalization caused a significant reduction in performance in general.

### 4.4 Spatial domain detection

For each method, in addition to the quantitative assessment using the five metrics, we visualized the spatial plot of gene expression of the top performing models by comparing the predicted and observed gene expression of the genes with the highest PCC scores, as shown in Figure S2. By observation, STimage, DeepSpaCE, and BLEEP effectively predicted the spatial gene expression patterns consistent with the ground truth measured by spatial transcriptomics.

When comparing to the H&E image, we broadly observed that the expression of these top performing genes correlated with the morphological features of the tissue. Such genes are likely suitable for use in applications that predict gene expression using images only.

Next, we evaluated whether the predicted gene expression profiles could be used to map spatial domains by downstream clustering analysis. We clustered the predicted gene expression and compared with those clusters obtained by using the observed (ground truth) gene expression values and with the pathological annotations provided by pathologists (Figure S3). We used the Wu *et al*. BC dataset, which had pathological annotation available (Wu *et al*. 2021). Specifically, we clustered the spatial spots using the predicted expression of the functionally relevant genes for each spot using the *K*-means clustering algorithm with *K* set to be the same as the number of cell types used in the pathologist annotation. We note that DeepSpaCE had a similar clustering approach using the predicted gene expression values from the model; however, this was not applied in the original work for other methods, and for purposes of fair comparison we applied the same clustering procedure to the raw outputs from each model.

Figure S3 reports the Adjusted Rand Index (ARI) of the clustering results obtained using the predicted gene expression profiles from the top four models, with reference to the pathologist annotations as the ground truth.

Interestingly, in some instances we observed that the clustering outcomes based on predicted gene expression aligned more closely with pathological annotations compared to those based on observed gene expression, as measured by the ARI. This result suggests that the predicted gene expression values could be useful even though not all genes can be predicted with high accuracy yet.

### 4.5 Integration with histopathological foundation model

**Figure 4:**
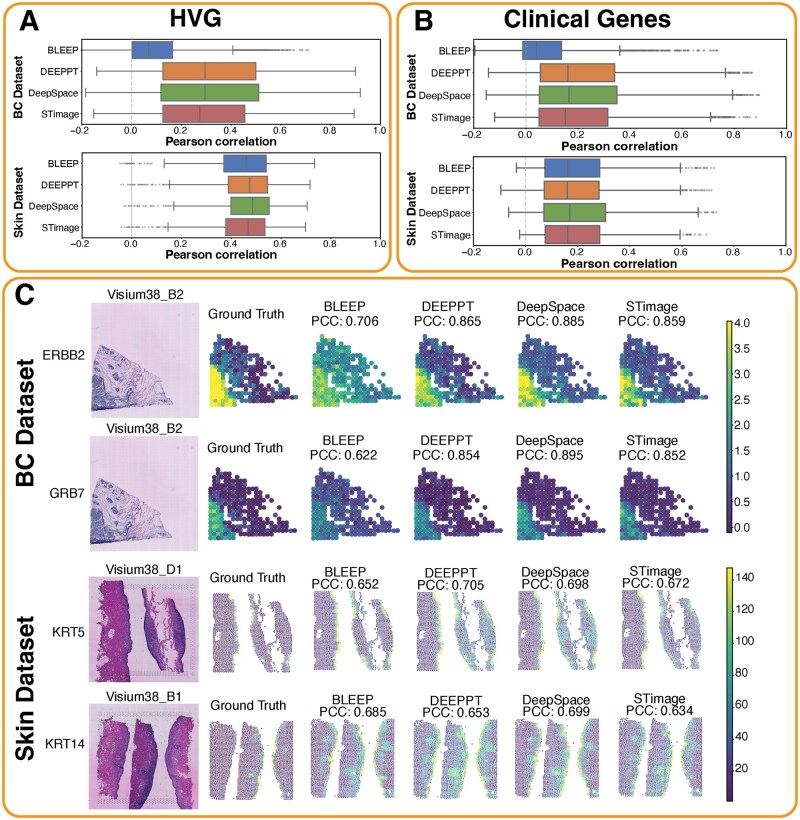
Performance evaluation of foundation model-integrated spatial gene expression prediction. Four gene expression prediction methods (BLEEP, DeepPT, DeepSpaCE, and STimage) were restructured to take pre-computed Virchow2 histopathology foundation model embeddings as input. (A, B) Quantitative performance assessment using Pearson correlation coefficients (PCC). Box plots show the correlation distribution for (A) highly variable genes (HVG) and (B) clinical genes across the breast cancer (BC) and skin datasets. The dashed line indicates a correlation of 0. (C) Visualization of spatial gene expression prediction. Spatial maps compare the ground-truth expression against predictions from the four models for representative marker genes (ERBB2, GRB7, KRT5, KRT14) on specific tissue sections. The PCC value for each prediction is annotated above the corresponding spatial plot.

Overall, the integration of the foundation model yielded higher predictive performance compared to the original model architectures. The mean values for PCC performance for HVG genes in breast cancer and skin cancer (shown in Figure 2) are lower than the values boosted by adding foundation models as shown in Figure 4. In the skin cancer dataset, we observed consistency in predictive capability, with all four models demonstrating similar performance levels. Conversely, in the breast cancer dataset, BLEEP showed the lowest performance in both the HVG and CRG tasks. Overall, the predictive accuracy for CRGs was generally lower than that for HVGs across the benchmarks (Figure 4A and B).

The spatial prediction patterns of key clinical genes were visually examined (Figure 4C). All four models successfully reconstructed the spatial expression patterns for ERBB2 and GRB7 in breast cancer tissue, as well as KRT5 and KRT14 in skin cancer tissue. However, BLEEP suffers from an inflation issue, tending to predict high gene expression levels in regions of low tissue density (e.g. adipose tissue) in the breast cancer dataset. It also shows lower PCC compared with other models, whereas the other models maintain tighter alignment with the ground-truth gene expression and tissue morphology.

## 5 Discussion

We systematically benchmarked seven existing methods to assess prediction accuracy and generalization in six datasets, four organs, two tissue types, two spatial resolutions, and three experimental protocols. We used five metrics and two gene sets (1000 highly variable genes and 1630 functionally relevant genes). Although performance varies between models, with some models consistently producing better performance, our comparison demonstrates that, on average, existing methods do not predict expression to a high degree of accuracy compared to the ground truth. The majority of genes are predicted by the top-performing models with an average Pearson correlation ranging between 0.1 and 0.3. We identified highly predictable genes that exhibit correlations exceeding the average correlation observed across the two full gene sets: the most variable genes and functionally relevant genes. Our findings suggest that selecting a subset of useful genes and focusing on a smaller, targeted gene set could improve performance while extracting relevant biological information and reducing computational costs. Furthermore, we showed that adding histopathological foundation models as a feature extractor markedly improves model performance.

We identified four top-performing methods, namely DeepPT, DeepSpaCE, BLEEP, and STimage that consistently ranked higher in the majority of comparisons for both in-domain and out-of-domain experiments over all metrics. Methods based on the transformer architecture (HisToGene, Hist2ST) and the early ST-net model did not perform as well. Notably, transformer-based models do not scale to a large number of spots/tiles. In the original papers, these models were developed and tested using the legacy Visium datasets, which contain only a few hundred spots per sample, but not for the more recent 10x Visium datasets that have several thousand spots per sample. To apply these models to 10x Visium datasets, we modified the data-loading strategies (see Algorithm Settings in the Supplementary Materials for details).

For out-of-domain experiments using breast cancer datasets, we found that technical variation between image and gene expression from Wu *et al*. and 10× Genomics had a significant impact on the performance. We suggest that proper data preprocessing is needed. We found that image augmentation, especially rotation, improved performance across all metrics. Variance stabilizing transformation methods, such as log transformation, appear to slightly improve the models’ overall performance. In contrast, library size normalization (referred to as log-norm, or norm in Table S2, where the total signal post normalization becomes the same post normalization) tended to reduce prediction accuracy. Regarding the effects of spatial resolution and sample sizes, from our experiments with the breast cancer OOD datasets, we did not observe substantial differences between the overall performance of the seven models when applying them to the high spatial resolution Visium (fewer samples) and low resolution Her2ST datasets.

The predictability of a gene is non-uniform and heavily influenced by its inherent expression characteristics and spatial distribution (Figures S5 and S6). We evaluated four spatial transcriptomics prediction methods (BLEEP, DEEPPT, DeepSpace, and STimage) against three ground-truth gene features: spatial autocorrelation (Moran’s *I*), mean expression magnitude, and sparsity (dropout fraction). Across all methods, genes with high Moran’s *I* values, indicating organized spatial patterning rather than stochastic distribution, exhibit a strong linear increase in Pearson correlation coefficients (PCC). This demonstrates that deep learning models effectively leverage visual features from the tissue microenvironment to predict morphologically structured transcripts. Similarly, highly expressed genes yield superior PCCs, likely due to a more favorable signal-to-noise ratio in the ground-truth Visium data that provides a stable target during model training. Conversely, an inverse relationship is observed between dropout fraction and predictability. In the skin dataset, prediction accuracy collapses as the dropout fraction approaches 1.0, confirming that ubiquitously detected genes provide continuous supervision signals necessary for model convergence. Notably, the breast cancer dataset exhibits high inter-sample variance (exemplified by the distinct sample clusters), which reflects pronounced inter-patient heterogeneity in tumor morphology. Ultimately, these benchmarks suggest that for downstream biological discovery, researchers should prioritize predictions for genes characterized by high Moran’s *I* and low dropout fractions to minimize the risk of analysing model-generated noise. For downstream analyses, our analysis of spatial domain detection indicates that predicted gene expression can be used to cluster regions of the image with results as consistent to pathological annotation as using the original spatial transcriptomics data. However, the varied quality of clusters indicates that care should be taken when interpreting these results.

In terms of scalability, we note that the transformer-based methods Hist2ST and HisToGene do not scale well to data with thousands of spots, such as the Visium data, due to the computation of global attention between spots.

In contrast, the other methods that treat spots as data points for mini-batch sampling are more scalable. Improvements in efficiency and scalability for deep learning methods should be considered for future developments and will be crucial as ST technology matures and the total number of spots and cells are rapidly increasing.

### 5.1 Limitations

Our benchmarking comparison shows that there is still a large gap between the performance of existing models and effectively usable outcomes. Overall performance remains constrained by relatively low absolute correlation, which is insufficient for reliable clinical or discovery applications. These models tend to perform well only for genes with strong and distinct spatial patterns, while struggling to capture more subtle or diffuse expression signals. Generalization is another major challenge, as performance often drops sharply when applied to entirely unseen tissue sections, indicating a strong dependence on large and diverse training datasets. In addition, many transcriptional changes do not manifest as immediate or visible morphological features in H&E images, making such genes inherently difficult or impossible to predict from histology alone. Finally, most current approaches rely on architectures adapted from standard computer vision models, which often lack appropriate spatial context modeling and may not be well suited to the unique structural and biological characteristics of histopathological data.

### 5.2 Conclusions and perspectives

Given that there is such a large component of uncertainty in the results of model predictions with many genes not being able to be predicted reliably by current methods, it is important that existing methods are informative of their own prediction uncertainty. Except for STimage, most existing methods do not quantify uncertainty in the model prediction, which is important to establish reliability and robustness when testing on new data (Abdar *et al*. 2021).

The rapidly evolving landscape of ST technology introduces additional complexities and variation (Du *et al*. 2023), with improvement in resolution and fidelity, together with an increase in sample sizes. This provides numerous challenges and opportunities to current deep learning methods that are adopted from domains with abundant and relatively uniform data, such as natural images. Increasing the available amount of data in the training process, and/or possibly utilizing *foundation models* trained on millions of image patches would likely better facilitate learning relationships that could generalize better and not be limited to smaller, cancer-specific domains.

Recent advances in histopathological foundation models have demonstrated a remarkable capability to capture complex morphological features (Chen *et al*. 2024, Xu *et al*. 2024, Zimmermann *et al*. 2024), leading to their increased deployment in tasks such as automated diagnosis, tumour grading, and molecular subtyping. These models have shown strong performance in cancer detection and the prediction of clinically relevant biomarkers, highlighting their potential to augment pathologist decision-making and improve diagnostic consistency. In this context, models that extend beyond traditional classification to infer underlying molecular signatures, such as gene expression from H&E stained images, represent a natural progression, offering biological insights from routinely collected slides without the need for costly molecular assays. However, while integrating these high-dimensional representations with gene expression regressors has yielded performance gains, predictive accuracy for clinical genes remains limited across current architectures. This suggests that enhanced image representations alone may not fully capture the subtle morphological signals linked with complex clinical pathways. To bridge this gap and reinforce the clinical applicability of this research, further refinements in multimodal fusion strategies and larger training datasets specifically tailored for spatial transcriptomics are essential.

## Supplementary Material

vbag202_Supplementary_Data

## Data Availability

The datasets analysed in this study are available here: (i) human HER2-positive breast tumor ST data https://github.com/almaan/her2st, (ii) 10× Genomics Visium data and Swarbrick’s Laboratory (Wu *et al*.) Visium data https://doi.org/10.48610/4fb74a9, (iii) the human cutaneous squamous cell carcinoma https://www.ncbi.nlm.nih.gov/geo/query/acc.cgi?acc=GSE144240, (iv) skin cancer melanoma dataset https://www.ncbi.nlm.nih.gov/geo/query/acc.cgi?acc=GSE221390, (v) kidney cancer dataset Raghubar *et al*. (2023), (vi) liver disease dataset https://www.ncbi.nlm.nih.gov/geo/query/acc.cgi?acc=GSE240429. We collected the six datasets and uploaded them to UQ eSpace, publicly accessible at https://doi.org/10.48610/3e7a235. The source code used to produce the results reported here is available at https://github.com/BiomedicalMachineLearning/DeepHis2Exp.
