# Supplementary material for: Performance of deep learning methods for spatial gene expression prediction from histology images: a comprehensive assessment
Source: Bioinform Adv. 2026 Jul 20;6(1):vbag202. doi: 10.1093/bioadv/vbag202 (PMC13445978; doi:10.1093/bioadv/vbag202)
Supplement: Supplementary file 1 [file vbag202_supplementary_data.zip › Supplementary_Figure_S1.pdf]

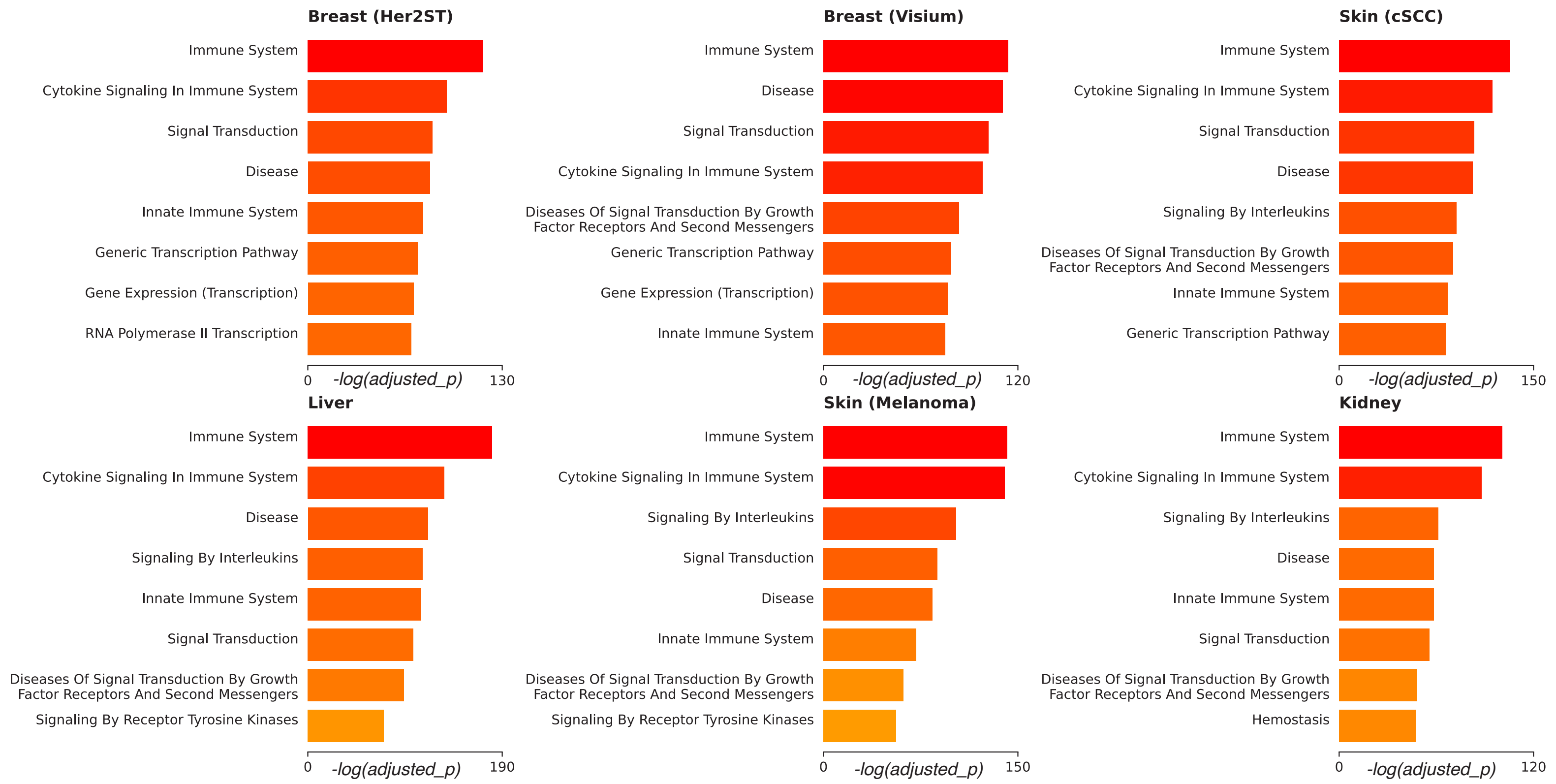

Breast (Her2ST)
Immune System
Cytokine Signaling In Immune System
Signal Transduction
Disease
Innate Immune System
Generic Transcription Pathway
Gene Expression (Transcription)
RNA Polymerase II Transcription
0
-log(adjusted_p)
130
Breast (Visium)
Immune System
Disease
Signal Transduction
Cytokine Signaling In Immune System
Diseases Of Signal Transduction By Growth Factor Receptors And Second Messengers
Generic Transcription Pathway
Gene Expression (Transcription)
Innate Immune System
0
-log(adjusted_p)
120
Skin (cSCC)
Immune System
Cytokine Signaling In Immune System
Signal Transduction
Disease
Signaling By Interleukins
Diseases Of Signal Transduction By Growth Factor Receptors And Second Messengers
Innate Immune System
Generic Transcription Pathway
0
-log(adjusted_p)
150
Liver
Immune System
Cytokine Signaling In Immune System
Disease
Signaling By Interleukins
Innate Immune System
Signal Transduction
Diseases Of Signal Transduction By Growth Factor Receptors And Second Messengers
Signaling By Receptor Tyrosine Kinases
0
-log(adjusted_p)
190
Skin (Melanoma)
Immune System
Cytokine Signaling In Immune System
Signaling By Interleukins
Signal Transduction
Disease
Innate Immune System
Diseases Of Signal Transduction By Growth Factor Receptors And Second Messengers
Signaling By Receptor Tyrosine Kinases
0
-log(adjusted_p)
150
Kidney
Immune System
Cytokine Signaling In Immune System
Signaling By Interleukins
Disease
Innate Immune System
Signal Transduction
Diseases Of Signal Transduction By Growth Factor Receptors And Second Messengers
Hemostasis
0
-log(adjusted_p)
120
