# Supplementary material for: Performance of deep learning methods for spatial gene expression prediction from histology images: a comprehensive assessment
Source: Bioinform Adv. 2026 Jul 20;6(1):vbag202. doi: 10.1093/bioadv/vbag202 (PMC13445978; doi:10.1093/bioadv/vbag202)
Supplement: Supplementary file 1 [file vbag202_supplementary_data.zip › Supplementary_Figure_S2.pdf]

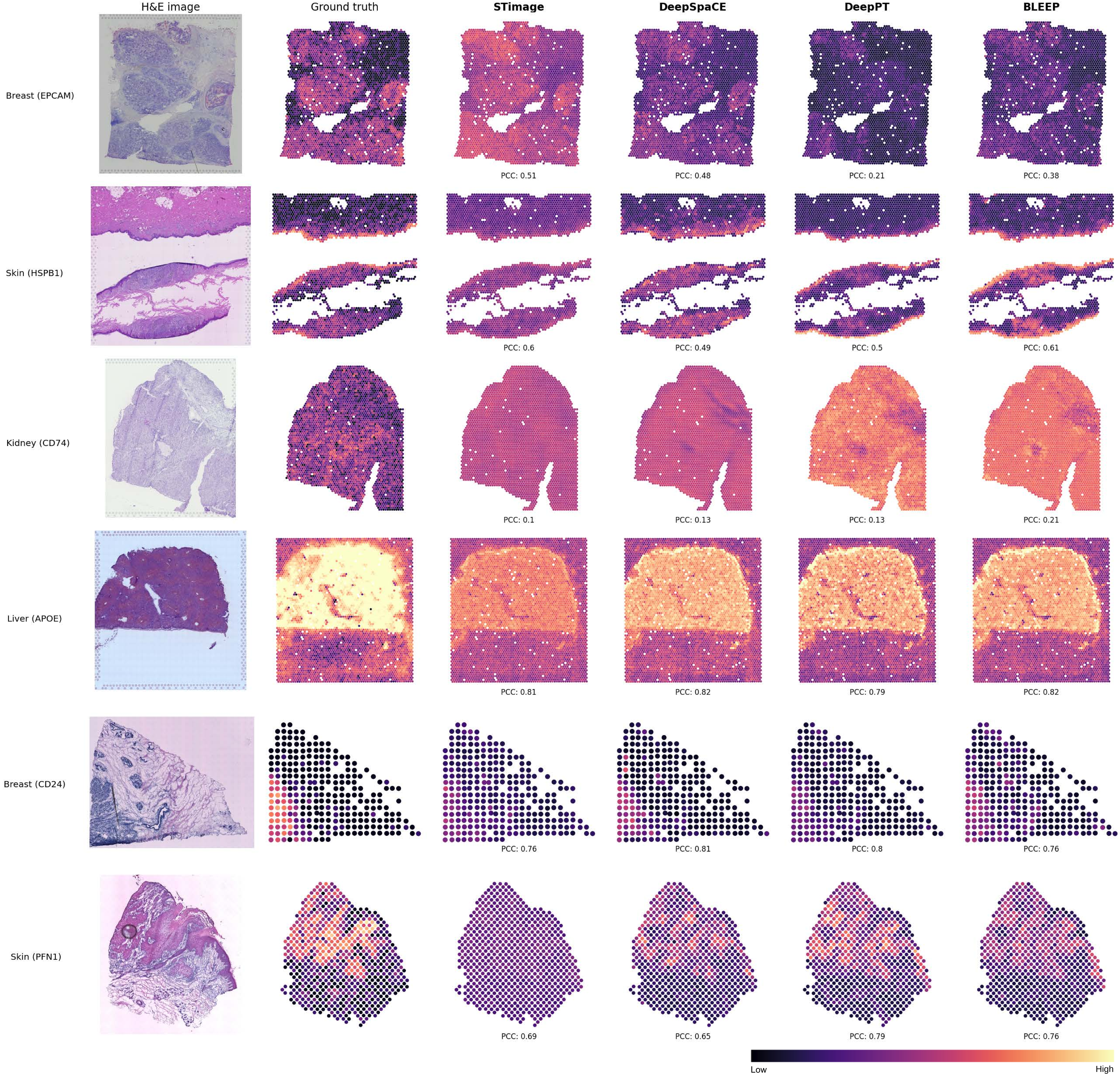

H&E image
Ground truth
STimage
DeepSpaCE
DeepPT
BLEEP
Breast (EPCAM)
PCC: 0.51
PCC: 0.48
PCC: 0.21
PCC: 0.38
Skin (HSPB1)
PCC: 0.6
PCC: 0.49
PCC: 0.5
PCC: 0.61
Kidney (CD74)
PCC: 0.1
PCC: 0.13
PCC: 0.13
PCC: 0.21
Liver (APOE)
PCC: 0.81
PCC: 0.82
PCC: 0.79
PCC: 0.82
Breast (CD24)
PCC: 0.76
PCC: 0.81
PCC: 0.8
PCC: 0.76
Skin (PFN1)
PCC: 0.69
PCC: 0.65
PCC: 0.79
PCC: 0.76
Low
High
