# Supplementary material for: Performance of deep learning methods for spatial gene expression prediction from histology images: a comprehensive assessment
Source: Bioinform Adv. 2026 Jul 20;6(1):vbag202. doi: 10.1093/bioadv/vbag202 (PMC13445978; doi:10.1093/bioadv/vbag202)
Supplement: Supplementary file 1 [file vbag202_supplementary_data.zip › Supplementary_Figure_S3.pdf]

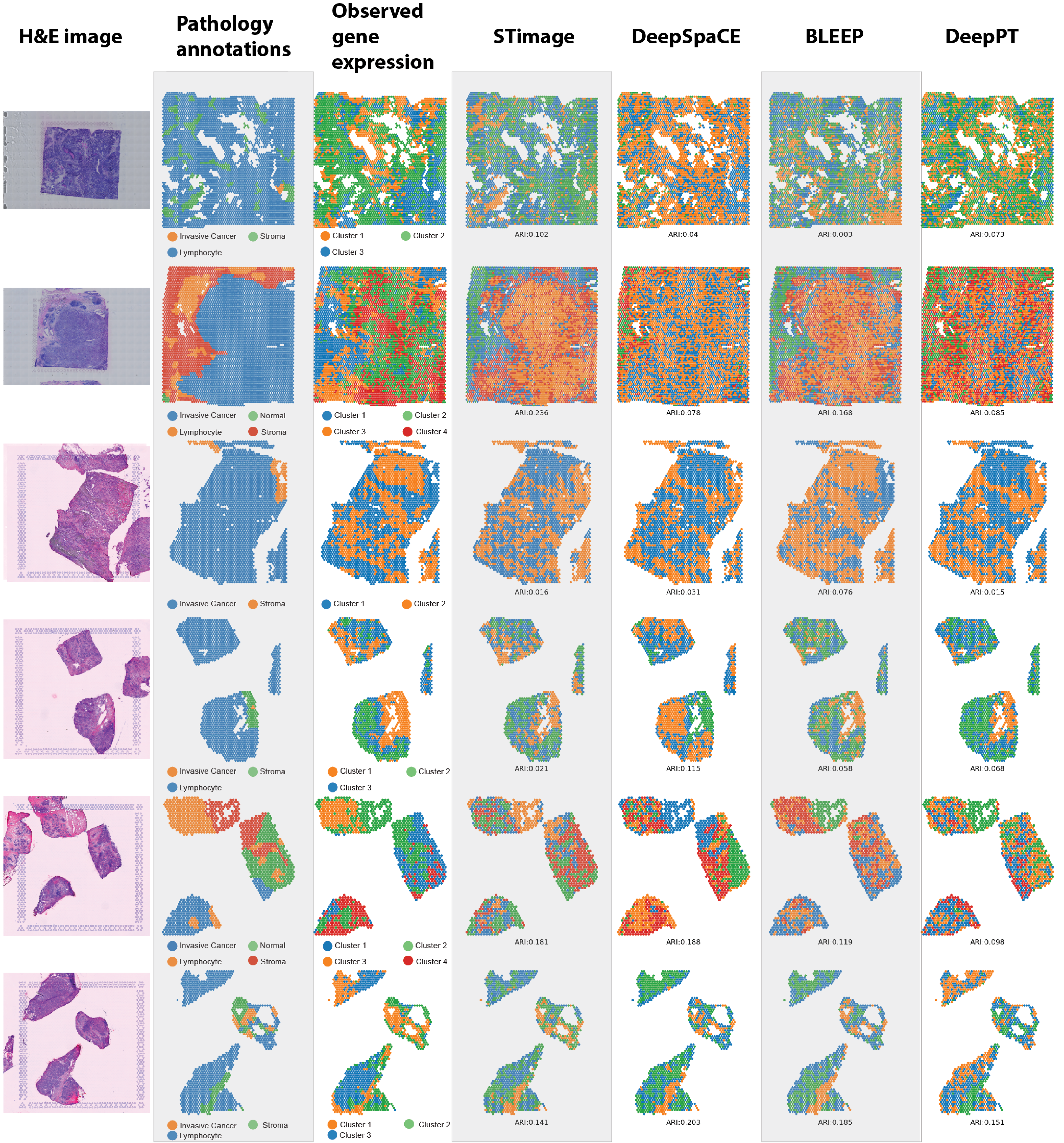
H&E image
Pathology annotations
Observed gene expression
STimage
DeepSpaCE
BLEEP
DeepPT
Invasive Cancer
Stroma
Lymphocyte
Cluster 1
Cluster 2
Cluster 3
ARI:0.102
ARI:0.04
ARI:0.003
ARI:0.073
Invasive Cancer
Normal
Lymphocyte
Stroma
Cluster 1
Cluster 2
Cluster 3
Cluster 4
ARI:0.236
ARI:0.078
ARI:0.168
ARI:0.085
Invasive Cancer
Stroma
Cluster 1
Cluster 2
ARI:0.016
ARI:0.031
ARI:0.076
ARI:0.015
Invasive Cancer
Stroma
Lymphocyte
Cluster 1
Cluster 2
Cluster 3
ARI:0.021
ARI:0.115
ARI:0.058
ARI:0.068
Invasive Cancer
Normal
Lymphocyte
Stroma
Cluster 1
Cluster 2
Cluster 3
Cluster 4
ARI:0.181
ARI:0.188
ARI:0.119
ARI:0.098
Invasive Cancer
Stroma
Lymphocyte
Cluster 1
Cluster 2
Cluster 3
ARI:0.141
ARI:0.203
ARI:0.185
ARI:0.151
