# Supplementary material for: Performance of deep learning methods for spatial gene expression prediction from histology images: a comprehensive assessment
Source: Bioinform Adv. 2026 Jul 20;6(1):vbag202. doi: 10.1093/bioadv/vbag202 (PMC13445978; doi:10.1093/bioadv/vbag202)
Supplement: Supplementary file 1 [file vbag202_supplementary_data.zip › Supplementary_Figure_S4.pdf]

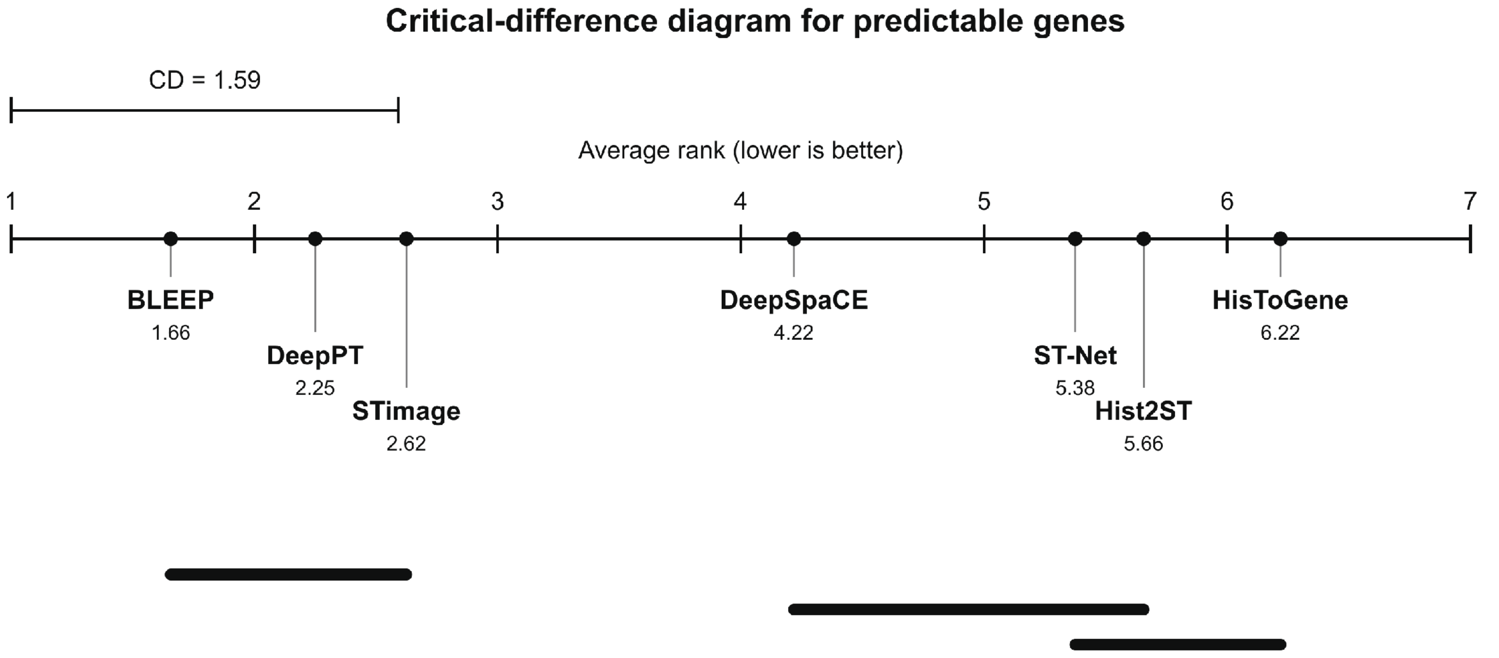
Critical-difference diagram for predictable genes
CD = 1.59
Average rank (lower is better)
1
2
3
4
5
6
7
BLEEP
1.66
DeepPT
2.25
STimage
2.62
DeepSpaCE
4.22
ST-Net
5.38
Hist2ST
5.66
HisToGene
6.22
