# Supplementary material for: Performance of deep learning methods for spatial gene expression prediction from histology images: a comprehensive assessment
Source: Bioinform Adv. 2026 Jul 20;6(1):vbag202. doi: 10.1093/bioadv/vbag202 (PMC13445978; doi:10.1093/bioadv/vbag202)
Supplement: Supplementary file 1 [file vbag202_supplementary_data.zip › Supplementary_Figure_S5.pdf]

BC Dataset

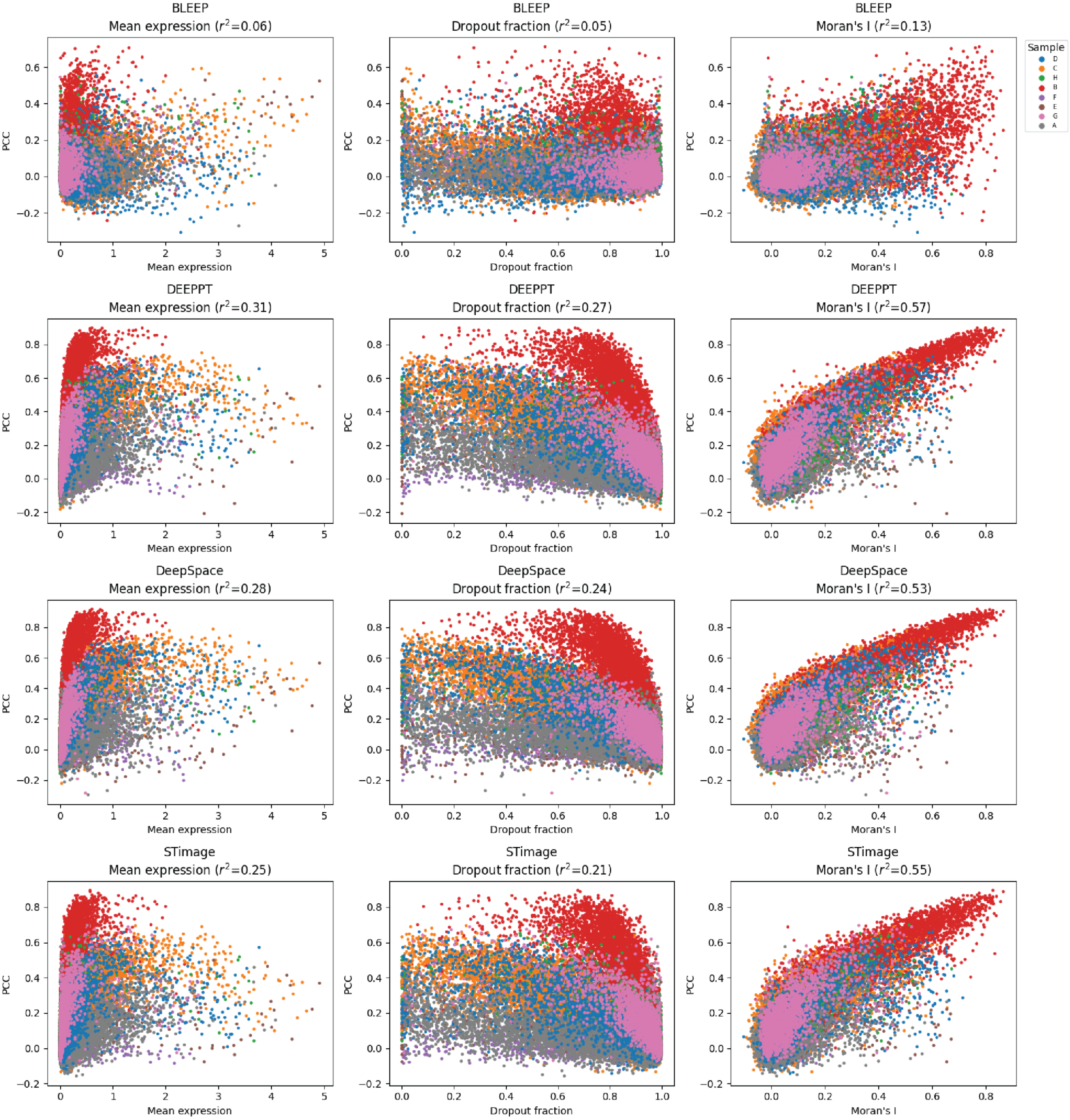

BLEEP
Mean expression (r^2=0.06)
BLEEP
Dropout fraction (r^2=0.05)
BLEEP
Moran's I (r^2=0.13)
DEEPPT
Mean expression (r^2=0.31)
DEEPPT
Dropout fraction (r^2=0.27)
DEEPPT
Moran's I (r^2=0.57)
DeepSpace
Mean expression (r^2=0.28)
DeepSpace
Dropout fraction (r^2=0.24)
DeepSpace
Moran's I (r^2=0.53)
STimage
Mean expression (r^2=0.25)
STimage
Dropout fraction (r^2=0.21)
STimage
Moran's I (r^2=0.55)
PCC
Mean expression
Dropout fraction
Moran's I
Sample
D
C
H
B
F
E
G
A
