# Supplementary material for: Performance of deep learning methods for spatial gene expression prediction from histology images: a comprehensive assessment
Source: Bioinform Adv. 2026 Jul 20;6(1):vbag202. doi: 10.1093/bioadv/vbag202 (PMC13445978; doi:10.1093/bioadv/vbag202)

Skin Dataset

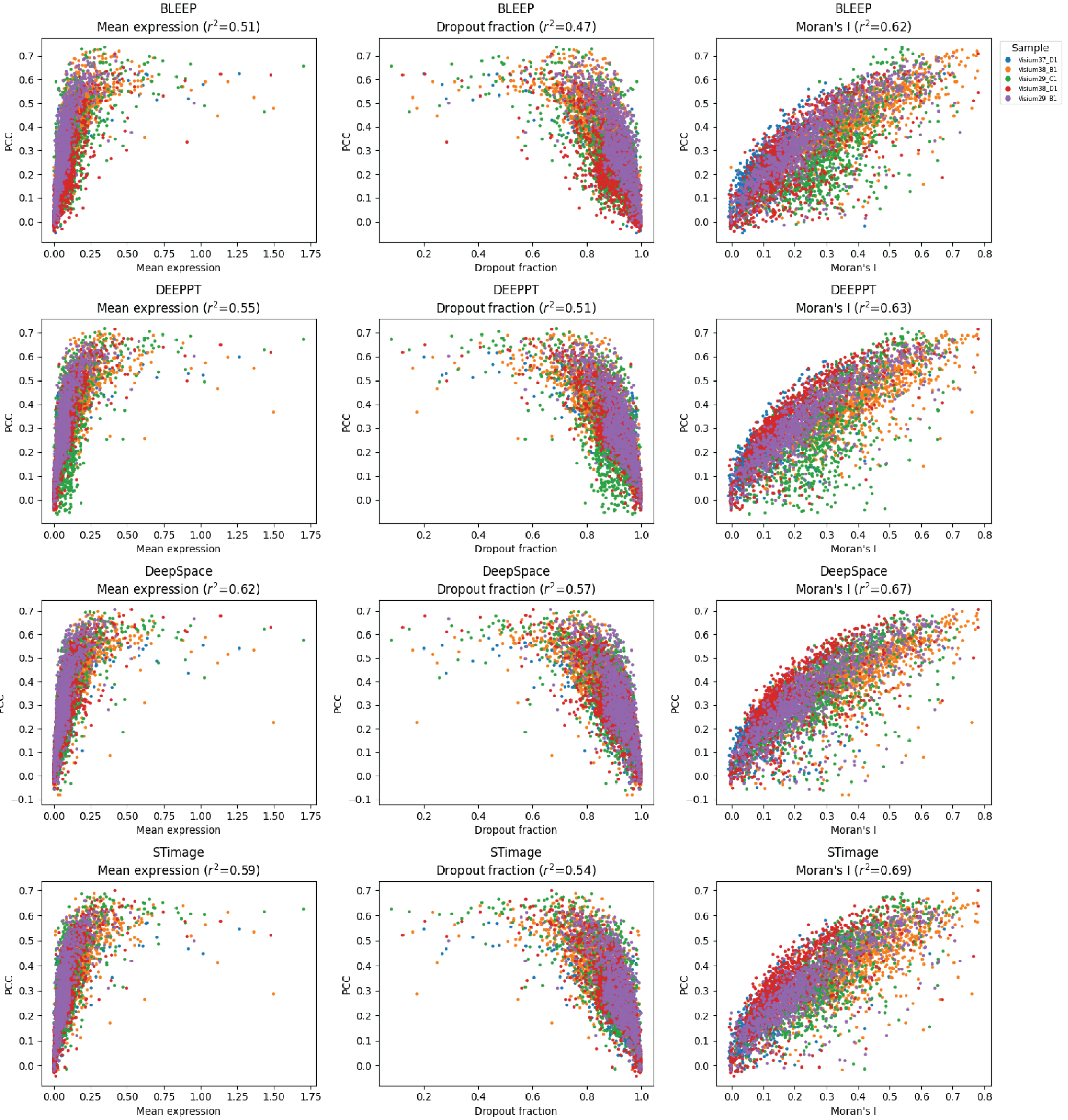

Supplement: Supplementary file 1 [file vbag202_supplementary_data.zip › Supplementary_Figure_S6.pdf]
