# Supplementary material for: Performance of deep learning methods for spatial gene expression prediction from histology images: a comprehensive assessment
Source: Bioinform Adv. 2026 Jul 20;6(1):vbag202. doi: 10.1093/bioadv/vbag202 (PMC13445978; doi:10.1093/bioadv/vbag202)
Supplement: Supplementary file 1 [file vbag202_supplementary_data.zip › Supplementary_Material.pdf]

Bioinformatics Advances, YEAR, pp. 1–12
DOI added during production
Advance Access Publication Date: Day Month Year
Supplementary Material

Supplementary Materials for "Performance of deep learning methods for spatial gene expression prediction from histology images: a comprehensive assessment"

Jacky Xie,[1,2,#] Xiao Tan,[1,3,#] Yuanhao Jiang,[1] Hao Nguyen,[1,3] Nan Ye[2,*] and Quan Nguyen[1,3] *

[1]Institute for Molecular Bioscience, The University of Queensland, 306 Carmody Road, 4072, QLD, Australia
[2]School of Mathematics and Physics, The University of Queensland, St Lucia, 4067, QLD, Australia
[3]QIMR Berghofer Medical Research Institute, 300 Herston Road, 4006, QLD, Australia

*Corresponding authors. quan.nguyen@uq.edu.au and nan.ye@uq.edu.au
# Equal contribution

Abstract

This document contains the supplementary materials for the above manuscript, including detailed descriptions of the benchmarked deep learning methods, dataset and preprocessing details, metric definitions, and supplementary tables (S1–S3) and figures (S1–S6).

Keywords Deep learning; Spatial transcriptomics; Histology images; Digital pathology; Benchmarking; Gene expression prediction

Supplementary Materials

Summary of identified deep learning methods

We summarize the specific model architectures and loss functions for each method.

ST-Net He et al. (2020) is a convolutional neural network (CNN) model that employs a DenseNet-121 pre-trained on ImageNet Deng et al. (2009) as an image encoder. The whole-slide histology images are divided into 224×224-pixel patches for each spot, which are transformed into features by the image encoder. The prediction head is a single fully connected output layer, which directly predicts the vector of gene expression. ST-Net is trained by minimizing the mean squared error (MSE).

HisToGene Pang et al. (2021) utilizes a modified vision transformer (ViT) model as an image encoder, which is able to incorporate the spatial relation of image spots. 112×112-pixel image patches are flattened and passed into a linear layer to produce the patch embeddings of dimension 1024. In addition, a learnable linear layer is used to map the spatial coordinates of each spot into a positional embedding vector of the same dimension. The patch embeddings and position embeddings are aggregated via summation and then input into eight multi-headed attention layers to generate latent embeddings. The prediction head is a single feed-forward layer that directly outputs the gene expression values. The model is trained to minimize the MSE between the predicted and observed values.

Hist2ST Zeng et al. (2022) consists of three main modules – Convmixer, Transformer, and Graph Convolutional Network (GCN) – to learn a global feature representation for all image spots on a single tissue section. 112×112-pixel patches are extracted around each spot. The initial image encoder is a Convmixer, a variant of CNN, that produces image features for each spot. The transformer module encodes spatial locations and fuses them with the image features, employing eight multi-head attention layers to learn global spatial dependencies. The aggregated features from the transformer module are then input into the GCN module to explicitly learn local spatial dependencies. The prediction head is a fully connected layer. In addition, there is a distribution module that estimates parameters for Zero-Inflated Negative Binomial (ZINB) Eraslan et al. (2019) distributions for each gene; this is learned by minimizing the negative log-likelihood. Self-distillation Ge et al. (2021) is used to learn from augmented image patches. The loss function combines the MSE from the prediction head, the ZINB loss, and the self-distillation loss.

STimage Tan et al. (2023) is a CNN model that uses a ResNet50 image encoder to extract features from 299×299-pixel image patches. The features are input into a distribution module, which consists of two fully connected layers that output the parameters of a Negative Binomial (NB) distribution for each gene. The whole model is trained by minimizing the negative log-likelihood. The expectation (mean) and variance of the Negative Binomial distribution for each gene are computed based on the estimated parameters of the distribution for that gene. These parameters are the probability of success parameter, p, and the shape parameter r.

STimage uses the distributions from an ensemble of such models to measure uncertainty. Moreover, the ensemble approach in STimage accounts for variation across independent training runs and improves performance and robustness.

DeepSpaCE Monjo et al. (2022) is a CNN model that utilizes the VGG16 architecture as an image encoder. Image patches of size 150%

© The Author(s) YEAR. Copyright and licence statements to be updated by the publisher during production.

times the original spot dimensions are extracted from the histology image. The prediction head consists of two fully connected layers for predicting the gene expression. The smooth L1 loss is computed using the predicted and observed values.

DeepPT Hoang et al. (2024) extracts features from 224×224-pixel image patches using the ResNet50 architecture. It then employs an autoencoder to further encode the extracted features. The latent output of the autoencoder is utilized as input for the downstream fully connected layers, with the last layer predicting gene expression, where each output value is for a gene or a gene group of similar median gene expression values. DeepPT is trained by minimizing both the MSE loss between the predicted and observed gene expression values and the autoencoder loss between the initial features and their reconstructions.

BLEEP Xie et al. (2023) learns a bimodal embedding for image and gene expression data. The process involves extracting features from the image tiles and expression vectors using separate encoders. The image encoder is a pre-trained ResNet50, while a fully connected network serves as the gene expression encoder. The image and gene expression features are then separately projected into image embeddings and expression embeddings, with a shared dimension of 256. Contrastive learning is employed to align the latent space for the two embeddings by minimizing the cross entropy. During inference, the image patches are mapped to embeddings by the encoder, and the k-nearest expression profiles from the reference dataset are selected based on their proximity, measured by Euclidean distance, to each patch in the joint embedding space. The predicted expression profiles for the query patches are a linear combination of the selected expression profiles.

Dataset Details

As shown in Table S3, there are two lower resolution (100 µm per spot) raw datasets and six raw datasets of a higher 55 µm resolution.

The lower-resolution datasets consist of the HER2+ breast tumour ST dataset Andersson et al. (2021) and the human cutaneous squamous cell carcinoma (cSCC) Ji et al. (2020) dataset.

- The Her2ST dataset comprises 36 tissue sections from eight HER2+ patients, with data generated using the initial (legacy) version of the ST protocol before the method was further developed by 10x Genomics to increase the resolution to 55 µm per spot. Among the 36 samples, there are cell type labels for eight tissues annotated by pathologists.
- The cSCC dataset contained 12 tissue sections obtained from 4 patients, with each patient having three sections. The 12 tissue sections of the cSCC dataset were measured by the 10x Visium platform. Each sample (whole slide image) within two datasets consists of around 300 to 600 spots, with each spot containing the measurements for 11,871 genes and 13,337 genes respectively.

The higher-resolution Visium datasets consist of the Wu et al. breast cancer dataset Wu et al. (2021), two 10x breast cancer datasets, skin melanoma dataset, kidney cancer dataset, and liver disease dataset.

- The three breast cancer datasets were described in the STimage paper Tan et al. (2023). We grouped them into a single combined dataset with nine breast cancer tissue samples taken from two fresh-frozen and one FFPE tissues. Each sample was measured on the 10x Visium platform and contained around 1300 to 4900 spots, with each spot having the measurements for up to 36,601 genes.
- The skin melanoma dataset includes five slides from ten patients. Each sample was measured on the 10x Visium platform and contains around 3000 spots, with each spot containing the measurements for up to 17,941 genes.
- The liver disease dataset Andrews et al. (2022, 2023) includes four slides from Primary Sclerosing Cholangitis (PSC) patients, a rare form of non-cancer liver disease. The four slides were consecutive 16-micrometre thick slices of human liver tissue from neurologically deceased donor livers, suitable for transplantation that were optimal cutting temperature embedded (OCT), frozen, sliced with a cryostat and imaged using the 10x Genomics Visium platform.
- The kidney data was downloaded from Raghubar et al. Raghubar et al. (2023). The Visium dataset includes 10 samples for six patients with high-risk clear cell renal cell carcinoma (ccRCC). The samples represent three groups, para-tumour (n = 2), low-grade (n = 4, G1-G2) and high-grade (n = 4, G3-G4) with a total of 16,166 spots. Samples were quickly frozen post surgery. For spatial transcriptomics, 8 µm cryosections were used.

Data Preprocessing and Augmentation

The default preprocessing and augmentation methods for each method are summarized in Table S1. Below, we provide a more detailed overview of the processing steps applied to both gene expression data and image data for each method.

ST-Net first transforms the total expression in each spot by adding a pseudo count and then applying a logarithmic transformation. Image patches centred on each spot are extracted, with dimensions of 224×224 pixels. During training, image augmentation techniques are utilized, including random rotations and flips at a 50% probability.

Table S1 Summary of data transformation methods used in the benchmarked algorithms.

Model	Tile size	Gene expression pre-processing	Data augmentation
ST-Net	224×224	Log-transformation	Random rotation & flipping
HisToGene	112×112	Normalization + log-transformation	Random rotation & flipping + color jitter
Hist2ST	112×112	Normalization + log-transformation	Random rotation & flipping + color jitter (self-distillation strategy)
STimage	299×299	Log-transformation	Color normalization (vahadane) + random one of flipping, cropping, noise addition, blurring, distortion, contrast adjustment, colour-shifting + remove tiles with low tissue coverage (< 70%)
DeepSpaCE	224×224	SCtransform + minmax scaling	Remove tiles with high RGB values
BLEEP	224×224	Normalization + log transformation	Random rotation & flip
DeepPT	224×224	Normalization	Random rotation

Table S2 Performance results from benchmarking data processing techniques. The DeepPT model was used as a reference model, and the results are averaged across LOOCV runs on the cSCC dataset.

	Pearson corr.		Spearman corr.		Moran's I		SSIM		Cosine sim.	
	Mean	Median	Mean	Median	Mean	Median	Mean	Median	Mean	Median
**Expression normalization**										
Raw	0.29	0.27	0.26	0.23	0.18	0.17	0.20	0.14	0.48	0.47
Log1p	**0.31**	**0.29**	**0.28**	**0.24**	**0.22**	**0.19**	**0.25**	**0.18**	**0.53**	**0.51**
Log-norm	0.15	0.14	0.15	0.14	0.11	0.09	0.12	0.09	0.44	0.41
Minmax	0.26	0.24	0.23	0.21	0.17	0.15	0.22	0.18	0.44	0.43
Norm	0.05	0.03	0.02	0.01	0.04	0.02	0.02	0.01	0.36	0.33
**Image transformation**										
None	0.31	0.29	0.28	0.24	0.22	0.19	0.25	0.18	0.53	0.51
Blur	0.31	0.31	0.28	0.25	0.21	0.19	0.24	0.19	0.53	0.52
Random grayscale	0.32	0.32	0.28	0.26	0.21	0.19	0.25	0.19	0.53	0.52
Random rotation	**0.33**	**0.32**	**0.29**	**0.26**	**0.23**	**0.21**	**0.25**	**0.21**	**0.54**	**0.52**

HisToGene first excludes genes expressed in fewer than 1,000 spots across all sections. For each spot, gene expression values are normalized by calculating the UMI count for each gene relative to the total UMI counts across all genes in that spot. This normalized value is then multiplied by 1,000,000 and log-transformed after adding a pseudocount. The image patches are of size 112×112 pixels, equivalent to the diameter of each spot in the HER2+ breast cancer dataset. Image augmentation is applied by random rotation, horizontal flip, and color jitter.

Hist2ST follows a similar preprocessing procedure for images and gene expression as in HisToGene. Unlike HisToGene, Hist2ST uses data augmentation in training, specifically, in the self-distillation strategy. This is done by creating five randomly perturbed image views of the original image patch. These augmented views are generated using random grayscale, rotation, and horizontal flip transformations.

STimage preprocesses the gene counts by log transformation after adding a pseudocount. Image patches of size 299×299 pixels are extracted from each spot. Stain normalization using the Vahadane method Vahadane et al. (2016) is conducted on the tiles to ensure colour distribution patterns are akin to a template image. Tiles with low tissue coverage (less than 70%) are removed. For training, image augmentation techniques are utilized, including random flipping, scaling, rotation, blurring, adding noise, and colour jitter.

DeepSpaCE preprocesses the data by filtering out spots with low UMI counts or a low number of measured genes. UMI counts are normalized using the SCTransform Hafemeister and Satija (2019) function from the Seurat package, and expression values are adjusted through min-max scaling. Images of size 224×224 pixels are used, and those with high mean RGB values, above 80%, are excluded. Image augmentation is performed using diverse transformations, including flipping, cropping, noise addition, blurring, distortion, contrast adjustment, and colour-shifting, to enhance the model's performance and adaptability.

The BLEEP workflow involves normalizing the expression levels of each spot by dividing them by the total count and applying a log transformation. Image patches with dimensions of 224×224 pixels are extracted around each spot, and during training, they are augmented by random flips and rotations to enhance the model's performance.

The DeepPT performs image embedding for tiles of size 224×224 pixels using ResNet50. To minimize staining variation (heterogeneity and batch effects), color normalization was applied to the tiles. The RNA expression data is rank normalized to values in [0,1] twice: gene-wise and patient-wise.

Details for metrics

We describe each metric used and their computation, for a gene with predicted expression values $\mathbf{x} = (x_1, \ldots, x_n)$ and observed expression values $\mathbf{y} = (y_1, \ldots, y_n)$ at the n spots in a sample. For $\mathbf{x}$, we use $\bar{x}, s_x, \sigma_x$ to denote the average, sample standard deviation, and standard deviation, of the values in $\mathbf{x}$. In addition, we use $z(\mathbf{x}) = \left(\frac{x_1-\bar{x}}{s_x}, \ldots, \frac{x_n-\bar{x}}{s_x}\right)$ to denote the standardized version of $\mathbf{x}$, and we use $r(\mathbf{x})$ to denote the vector containing the ranks of $x_1, x_2, \ldots, x_n$. Similar notations are used for $\mathbf{y}$, and σ_{xy} denotes the covariance of $\mathbf{x}$ and $\mathbf{y}$. The formula for the five metrics are given below, with explanations for some further notations and the meanings of these metrics given in the following paragraphs.

$$\mathrm{PCC} = \frac{1}{n-1} z(\mathbf{x})^\top z(\mathbf{y}) = \frac{1}{n-1} \sum_i \frac{x_i - \bar{x}}{s_x} \frac{y_i - \bar{y}}{s_y}$$

$$\mathrm{SPC} = \mathrm{PCC} \text{ of } r(\mathbf{x}) \text{ and } r(\mathbf{y})$$

$$\mathrm{CSIM} = \frac{\mathbf{x}^\top \mathbf{y}}{\|\mathbf{x}\| \|\mathbf{y}\|}$$

$$\mathrm{SSIM} = \frac{(2\bar{x}\bar{y} + C_1) \cdot (2\sigma_{xy} + C_2)}{(\bar{x}^2 + \bar{y}^2 + C_1) \cdot (\sigma_x^2 + \sigma_y^2 + C_2)}$$

$$\text{Moran's I} = \frac{1}{n-1} z(\mathbf{x})^\top s(\mathbf{y})$$

Note that the standard Moran's I for measuring the spatial autocorrelation of the observed values $\mathbf{y}$ is simply $\frac{1}{n-1} z(\mathbf{y})^\top s(\mathbf{y})$.

All these metrics are correlation measures and do not depend on the absolute scale of the values, because the gene expression values are relative values, which makes the commonly used mean squared error or mean absolute error not a suitable performance metric when the predictions are just off by a scaling factor. PCC is one of the most commonly used correlation measuresering performance of observed and predicte, but it is sensitive to outliers. SPC measures the PCC between the ranks of the measured values, and is thus more robust against outliers. The cosine similarity is similar to PCC — in fact, PCC of $\mathbf{x}$ and $\mathbf{y}$ is simply the cosine similarity of the normalized $\mathbf{x}$ and $\mathbf{y}$. SSIM measures how well $\mathbf{x}$ and $\mathbf{y}$ are linearly correlated and whether they share the same means and variances. The variables C_1 and C_2 are constants used for stabilizing the division, with default values 0.0001 and 0.0009 respectively. These metrics do not account for any spatial relationship between data points, and our new Moran's I metric measures how well the predictions capture the spatial autocorrelations in the observed gene expression values.

Specifically, this involves first constructing a neighborhood graph for the n spots, where two spots i and j are neighbors if their Voronoi cells touch each other. Then we performed spatial smoothing on the standardized observed values $z(\mathbf{y})$ by computing the average neighbor spot value for each spot to obtain $s(\mathbf{y}) = (\tilde{y}_1, \ldots, \tilde{y}_n)$.

The inner product $z(\mathbf{x})^\top s(\mathbf{y})$ measures the amount of spatial autocorrelation captured in the predictions. A more explicit formula for $z(\mathbf{x})^\top s(\mathbf{y})$ is

$$\frac{1}{n-1} \sum_i \sum_j w_{ij} \frac{x_i - \bar{x}}{s_x} \frac{y_j - \bar{y}}{s_y},$$

where w_{ij} is the (i, j)-entry of the row-normalized adjacency graph for the spots. Specifically, $w_{ij} > 0$ if the Voronoi cells of spots i and j touch each other, and for each i, $\sum_j w_{ij} = 1$ with all positive w_{ij}'s being equal. Intuitively, the above Moran's I metric is the inner product between the standardized x vector and a spatially smoothed version of the standardized y vector, divided by $n - 1$.

Details for functionally relevant gene set

We consider a set of 1630 FGs consisting of genes that are involved in disease-relevant pathways, genes that correspond to cell-type markers, and the immunology and cancer markers for the examined tissue types. These genes were the intersection from well curated spatial transcriptomics gene panels, including 1253 genes from human pan-cancer panel and 1056 genes from human immunology panel (10x Genomics). The pan-cancer panel contains: 1) 21 cancer-related pathways such as Hedgehog-, Ras-, p53-, MAPK-, Notch-, mTOR-, TNF-, TGF-beta-, TLR-, and Wnt signaling pathways; 2) markers for 28 cancer cell/tissue types, such as colon cancer, breast cancer; 3) 17 cancer-related cellular processes such as immune response, chemotaxis; and 4) 37 functional annotations (e.g., nucleus, extracellular matrix) and processes (e.g., angiogenesis, mitosis). The immunology panel contains: 1) 20 immune pathways, 2) markers for 29 cell/tissue types (e.g., liver, lung, NK cells, macrophage, DC), 3) 41 immune-related cellular processes (e.g., inflammatory responses, innate immune responses), 4) 25 immune-related functional processes. Although these lists are not exhaustive and they evolve overtime, it is necessary to assess the prediction of functionally relevant genes, not just focusing on 1000 highly variable genes as previous reports.

Details for foundation model experiment

The input resolution for our experiment was strictly controlled to match the pretraining specifications of Virchow2. The model input tiles of 224 × 224 pixels at 0.5 microns per pixel (mpp) centered on each spatial transcriptomics spot, which corresponds to 20x magnification. To maximize the information transfer to the gene expression prediction models, we adopted the concatenation strategy. Let $T \in \mathbb{R}^{257\times1280}$ be the output tensor from the final layer of the Virchow2 encoder, where 1280 is the embedding dimension of the ViT-H architecture. $t_{cls} = T \in \mathbb{R}^{1280}$ represents the global class token. $T_{patches} = T[1 : 257] \in \mathbb{R}^{256\times1280}$ represents the sequence of patch tokens. We compute the mean patch token: $t_{mean} = \frac{1}{256}\sum_{i=1}^{256} T_{patches}[i] \in \mathbb{R}^{1280}$. The final feature vector x_{emb} utilized by our downstream models is the concatenation of these two vectors:

$$x_{emb} = [t_{cls}||t_{mean}] \in \mathbb{R}^{2560}$$

The benchmarking was done on two datasets – skin cancer and breast cancer – targeting two specific gene lists: the top 1000 Highly Variable Genes (HVG) and a clinically-relevant genes set (CRG). The CRG panel was systematically curated from the literature to capture biomarkers direct translational relevance, across three functional domains (i) diagnostic classification and lineage assignment (e.g. hormone receptors ESR1/PGR/HER2/ERBB2, squamous markers KRT5/KRT14), (ii) prognostic and risk stratification signatures (e.g. MammaPrint 70-gene signature, tumor suppressors TP53/PTEN), and (iii) therapy selection and resistance pathways (e.g. endocrine therapy, HER2-directed treatment, PARP and PI3K/AKT/mTOR inhibitors, FGFR/TRK inhibitors and immune checkpoint blockade).

Computational Training Settings

All the methods were first trained and tested with their default hyperparameters, pre-and post-processing steps, and data augmentation methods. We applied early stopping during training.

Each model was trained on a single NVIDIA V100 GPU with 32 GB RAM.

We additionally modified the training strategy for Hist2ST and HisToGene. Hist2ST and HisToGene were originally trained on Visium low-resolution data (Visium legacy). They employ transformer and graph neural network modules that require the loading and processing of all the spots in tissue samples at once. When training these methods with the Visium high-resolution data, which often contains more than 2000 spots per sample (i.e. 2000 image tiles), we faced out-of-memory issues. To address this issue,

we randomly sampled a few hundred spots without replacement to input into one mini-batch and the whole mini-batch is divided into ten more mini-batches for one training instance. The size of the mini-batch depends on the number of spots within the slides and computational resources.

Table S3 Summary of the datasets used in this paper. We combine the three BC visium datasets into one BC dataset and label them as a single dataset in the table, thus we have a total of six datasets in our experiments. HVG: Highly Variable Genes; FG: Functionally Relevant Gene; PG: Predictable Genes.

No.	Dataset	Samples	Tissues	Resolution	Spots	Genes	HVG (PG)	FG (PG)
1	Her2ST He et al. (2020)	36	Frozen	100 µm	13,620	11,871	785 (149)	1191 (373)
2	Wu et al. BC Visium Wu et al. (2021)	6	Frozen	55 µm	16,456	36,601	1000 (64)	1630 (344)
	10x BC Visium 10x Genomics (a)	2	Frozen	55 µm	7,785	36,601	1000 (64)	1630 (344)
	10x BC Visium 10x Genomics (b)	1	FFPE	55 µm	2,518	17,943	1000 (64)	1630 (344)
3	cSCC Ji et al. (2020)	12	Frozen	100 µm	8,209	13,337	134	1381 (419)
4	Melanoma Visium (Ours)	5	FFPE	55 µm	15,209	17,941	1000 (70)	1210 (366)
5	Liver Visium Andrews et al. (2022, 2023)	4	Frozen	55 µm	19,968	36601	1000 (408)	981 (471)
6	Kidney Visium Raghubar et al. (2023)	6	Frozen	55 µm	16,166	31915	987 (42)	1704 (247)

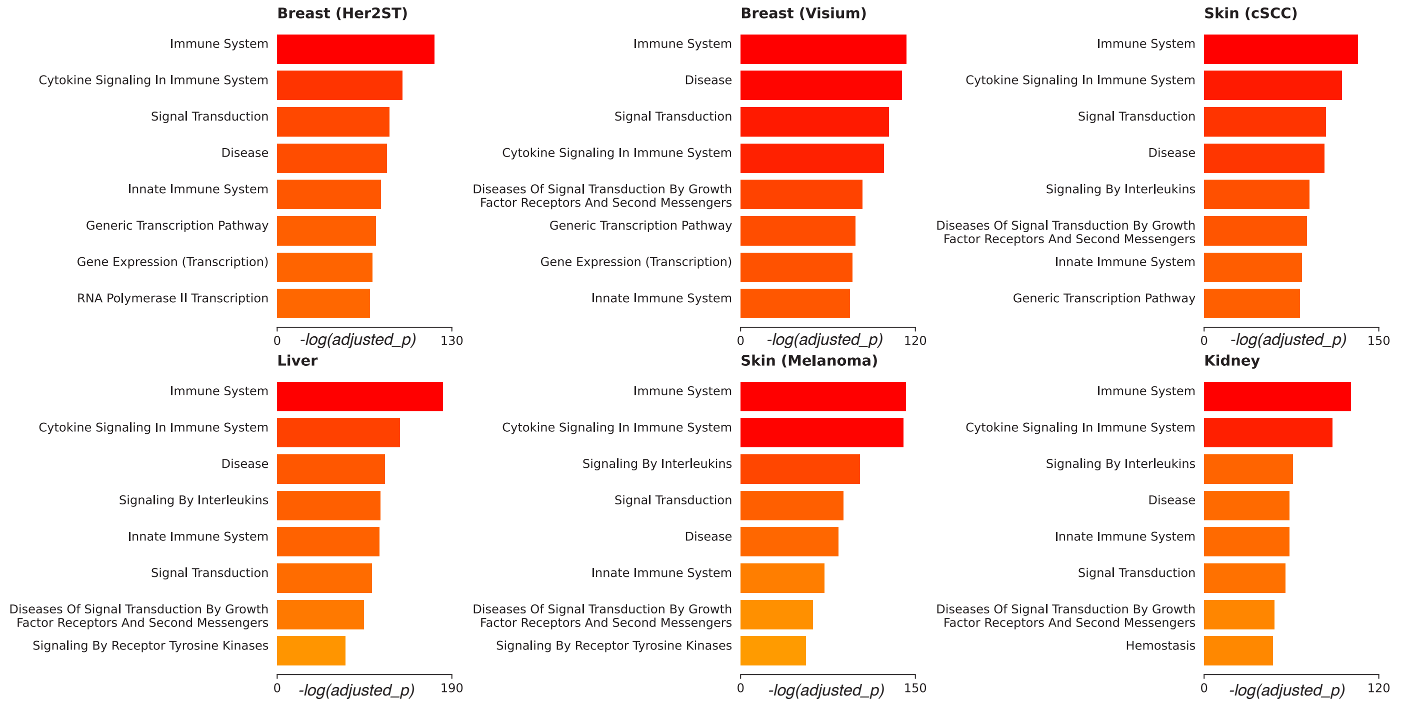

Figure S1 Gene enrichment analysis of predictable genes for each dataset, using the Reactome 2022 database. Bars display $-\log(p)$ of the adjusted p-value.

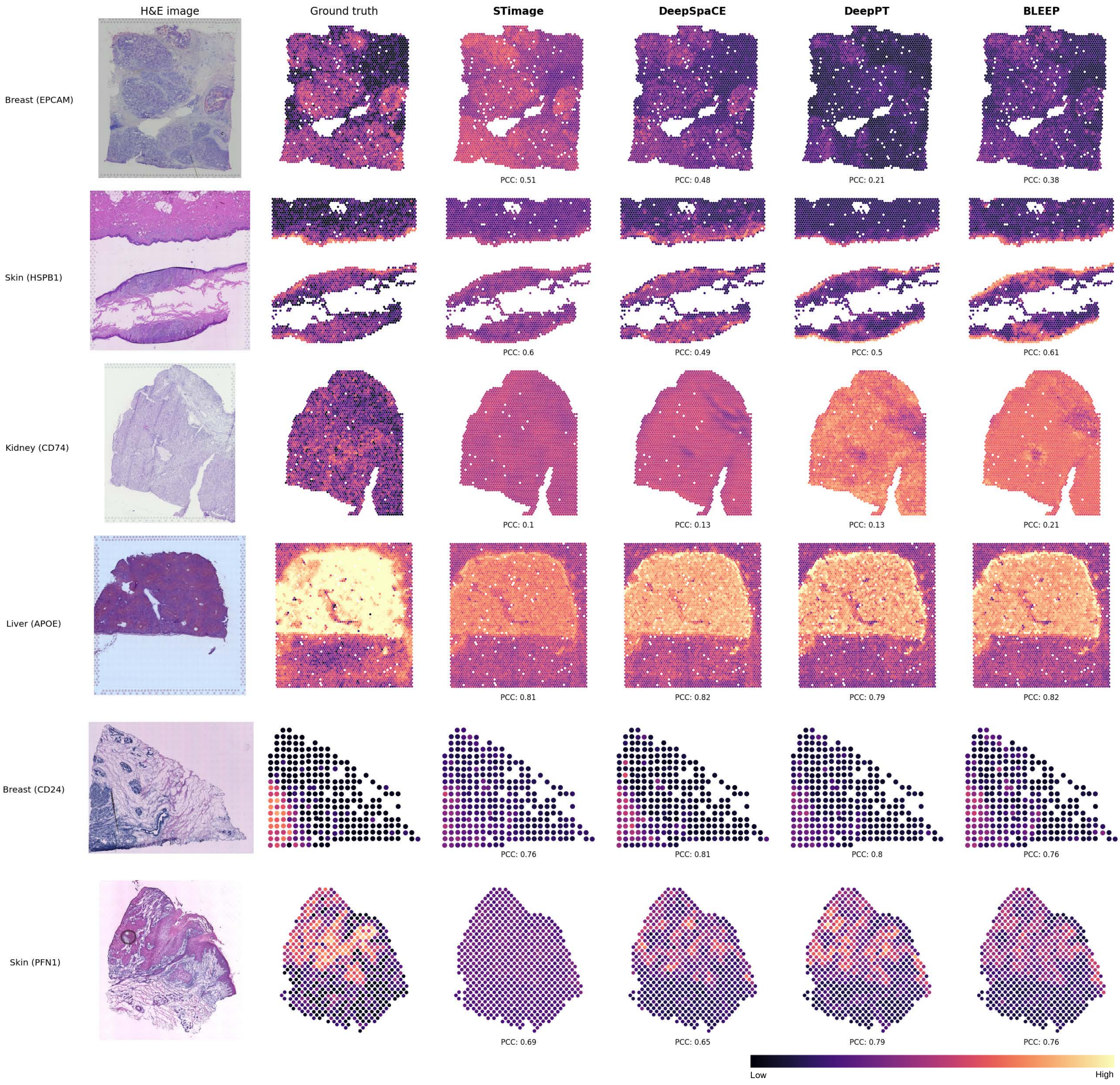

Figure S2 Visualization of the gene expression of the most predictable genes, selected by ranking the median Pearson correlation across all models for each dataset (organ). The columns show the predictions from the top 4 models. We note that the ground-truth measurement for the liver sample shows noisy signal outside the tissue region.

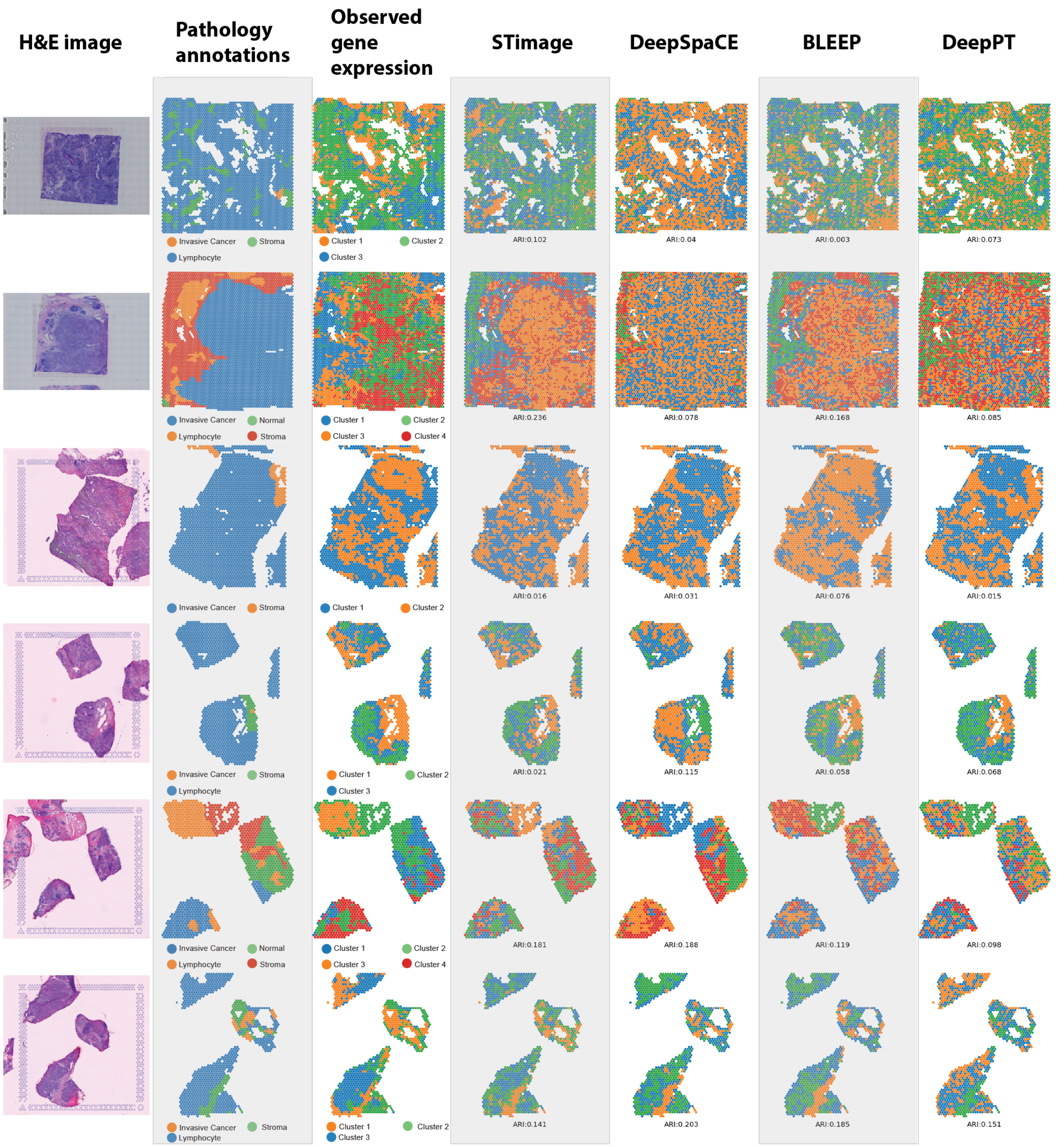

Figure S3 Clustering performance of observed and predicted gene expression on Wu et al. BC dataset. Visualization of clustering results, comparing the clusters using predicted gene expression values with pathological annotation (second column) and the clusters from using observed gene expression (third column). The pathological annotation of the the H&E images based on morphological features had four main categories including normal, stromal, lymphocyte aggregates, and invasive cancer. The genes used for clustering are the functionally relevant gene list.

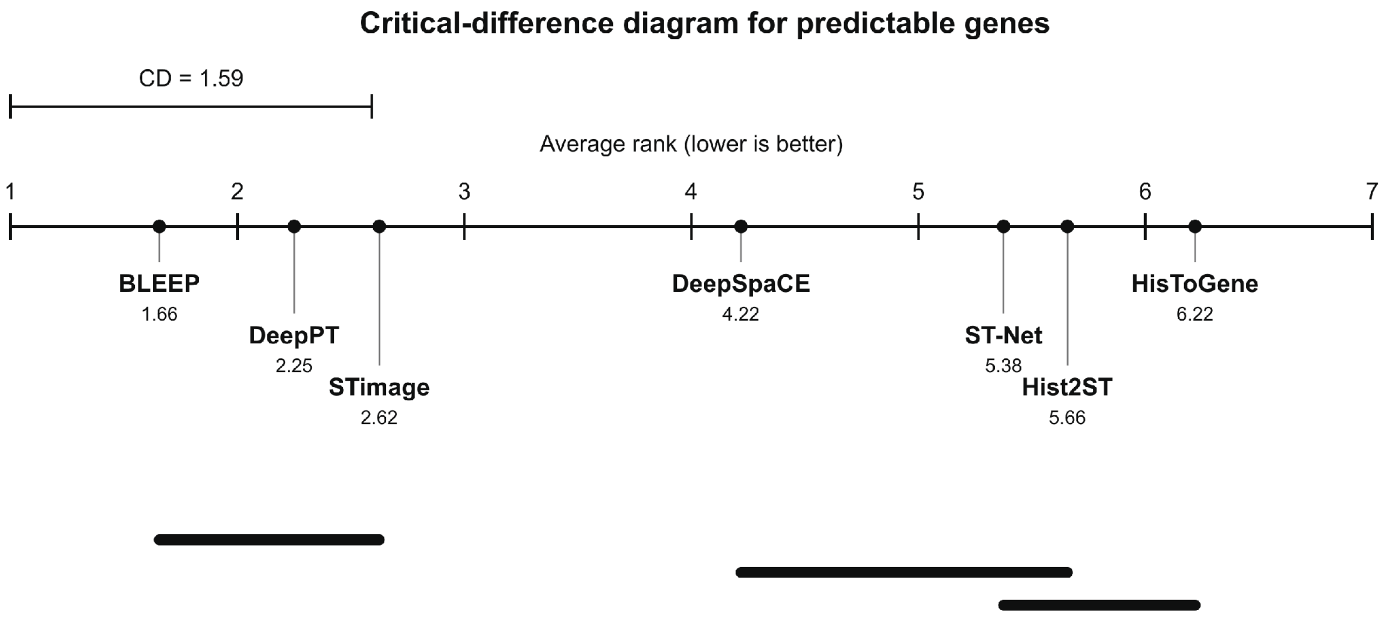

Figure S4 Critical-difference diagram comparing the seven methods on predictable genes. The critical difference was 1.59 at $\alpha = 0.05$. Black horizontal bars connect consecutive methods that were not significantly different according to the Nemenyi test. BLEEP, DeepPT, and STimage formed the leading group and were not significantly different from one another.

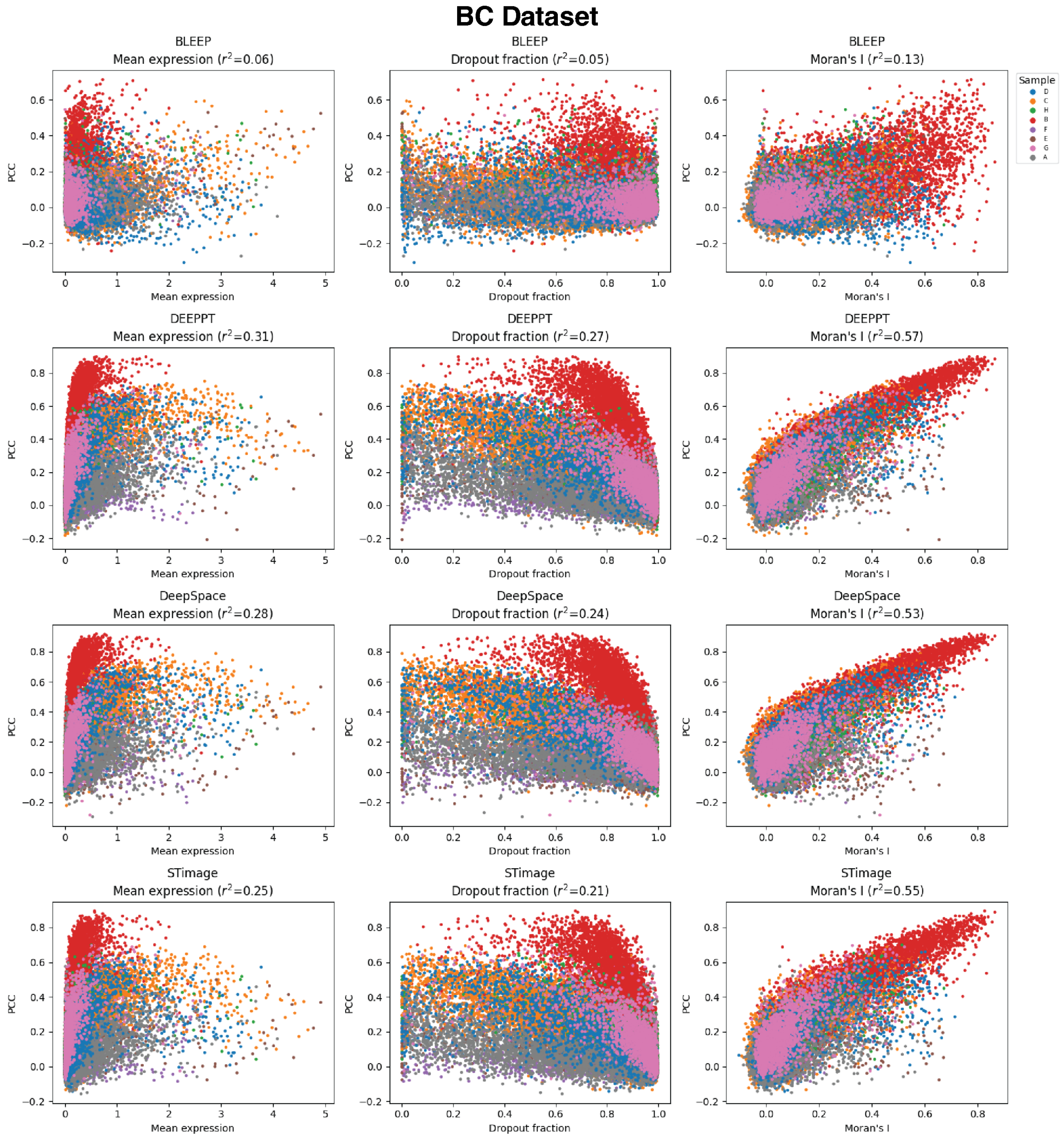

Figure S5 Evaluation of per-gene prediction accuracy correlates with expression level, dropout, and spatial autocorrelation in breast cancer. Scatter plots showing per-gene prediction accuracy (PCC) across four spatial transcriptomics prediction methods (rows) against three gene-level properties (columns) in breast cancer dataset. Gene properties (mean expression, dropout fraction, and Moran's I spatial autocorrelation) were computed per sample from the ground-truth expression matrix. Each point represents a single gene within a specific sample, color-coded by sample ID.

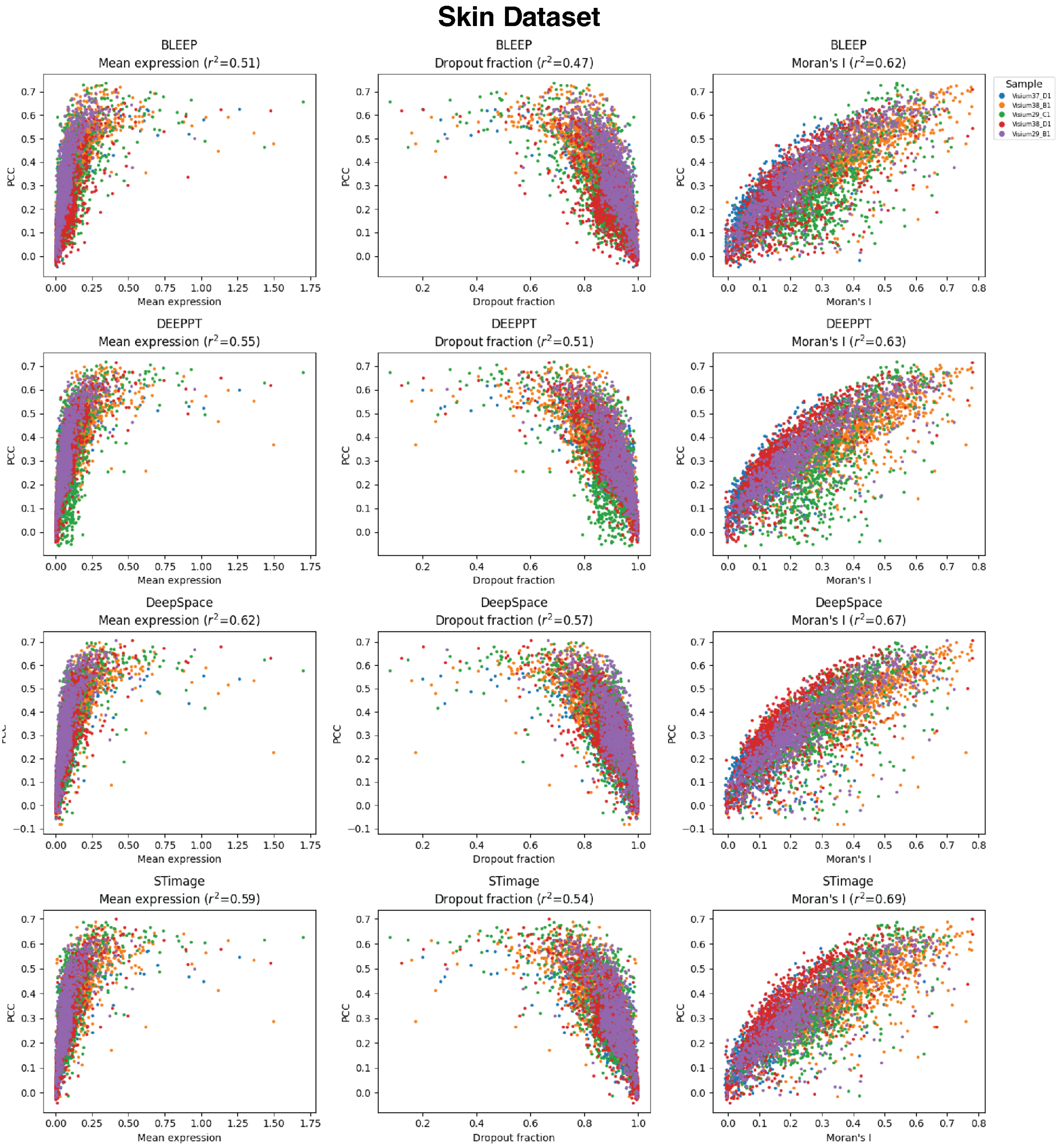

Figure S6 Evaluation of Per-gene prediction accuracy correlates with expression level, dropout, and spatial autocorrelation in skin tissue. Evaluation of prediction methods across the skin dataset, formatted as described in Figure S5

References

10x Genomics. High resolution mapping of the breast cancer tumor microenvironment using integrated single cell, spatial and in situ analysis of FFPE tissue, a. https://www.10xgenomics.com/products/xenium-in-situ/preview-dataset-human-breast.

10x Genomics. Human Breast Cancer: Ductal Carcinoma In Situ, Invasive Carcinoma (FFPE), b. https://www.10xgenomics.com/datasets/human-breast-cancer-ductal-carcinoma-in-situ-invasive-carcinoma-ffpe-1-standard-1-3-0.

A. Andersson, L. Larsson, L. Stenbeck, F. Salmén, A. Ehinger, S. Z. Wu, G. Al-Eryani, D. Roden, A. Swarbrick, Å. Borg, et al. Spatial deconvolution of her2-positive breast cancer delineates tumor-associated cell type interactions. *Nature communications*, 12 (1):6012, 2021.

T. S. Andrews, J. Atif, J. C. Liu, C. T. Perciani, X.-Z. Ma, C. Thoeni, M. Slyper, G. Eraslan, A. Segerstolpe, J. Manuel, et al. Single-cell, single-nucleus, and spatial RNA sequencing of the human liver identifies cholangiocyte and mesenchymal heterogeneity. *Hepatology Communications*, 6(4):821–840, 2022.

T. S. Andrews, D. Nakib, C. Perciani, X. Z. Ma, L. Liu, E. Winter, D. Camat, S. Chung, J. Manuel, S. Mangroo, et al. Single-cell and spatial transcriptomics reveals the human liver immunological landscape and myeloid dysfunction in psc. *bioRxiv*, pages 2023–07, 2023.

J. Deng, W. Dong, R. Socher, L.-J. Li, K. Li, and L. Fei-Fei. ImageNet: A large-scale hierarchical image database. In *2009 IEEE Conference on Computer Vision and Pattern Recognition*, pages 248–255. Ieee, 2009.

G. Eraslan, L. M. Simon, M. Mircea, N. S. Mueller, and F. J. Theis. Single-cell RNA-seq denoising using a deep count autoencoder. *Nature Communications*, 10(1):390, 2019.

Y. Ge, X. Zhang, C. L. Choi, K. C. Cheung, P. Zhao, F. Zhu, X. Wang, R. Zhao, and H. Li. Self-distillation with batch knowledge ensembling improves imagenet classification. *arXiv preprint arXiv:2104.13298*, 2021.

C. Hafemeister and R. Satija. Normalization and variance stabilization of single-cell rna-seq data using regularized negative binomial regression. *Genome biology*, 20(1):296, 2019.

B. He, L. Bergenstråhle, L. Stenbeck, A. Abid, A. Andersson, Å. Borg, J. Maaskola, J. Lundeberg, and J. Zou. Integrating spatial gene expression and breast tumour morphology via deep learning. *Nature biomedical engineering*, 4(8):827–834, 2020. Code: https://github.com/bryanhe/ST-Net.

D.-T. Hoang, G. Dinstag, E. D. Shulman, L. C. Hermida, D. S. Ben-Zvi, E. Elis, K. Caley, S.-J. Sammut, S. Sinha, N. Sinha, C. H. Dampier, C. Stossel, T. Patil, A. Rajan, W. Lassoued, J. Strauss, S. Bailey, C. Allen, J. Redman, T. Beker, P. Jiang, T. Golan, S. Wilkinson, A. G. Sowalsky, S. R. Pine, C. Caldas, J. L. Gulley, K. Aldape, R. Aharonov, E. A. Stone, and E. Ruppin. A deep-learning framework to predict cancer treatment response from histopathology images through imputed transcriptomics. *Nature Cancer*, 5(9):1305–1317, Sept. 2024. ISSN 2662-1347. doi: 10.1038/s43018-024-00793-2. URL `https://www.nature.com/articles/s43018-024-00793-2`.

A. L. Ji, A. J. Rubin, K. Thrane, S. Jiang, D. L. Reynolds, R. M. Meyers, M. G. Guo, B. M. George, A. Mollbrink, J. Bergenstråhle, et al. Multimodal analysis of composition and spatial architecture in human squamous cell carcinoma. *Cell*, 182(2):497–514, 2020.

T. Monjo, M. Koido, S. Nagasawa, Y. Suzuki, and Y. Kamatani. Efficient prediction of a spatial transcriptomics profile better characterizes breast cancer tissue sections without costly experimentation. *Scientific Reports*, 12(1):4133, 2022. https://github.com/tmonjo/DeepSpaCE.

M. Pang, K. Su, and M. Li. Leveraging information in spatial transcriptomics to predict super-resolution gene expression from histology images in tumors. *bioRxiv*, pages 2021–11, 2021. Code: https://github.com/maxpmx/HisToGene.

A. M. Raghubar, N. A. Matigian, J. Crawford, L. Francis, R. Ellis, H. G. Healy, A. J. Kassianos, M. S. Ng, M. J. Roberts, S. Wood, et al. High risk clear cell renal cell carcinoma microenvironments contain protumour immunophenotypes lacking specific immune checkpoints. *NPJ Precision Oncology*, 7(1):88, 2023.

X. Tan, O. Mulay, S. MacDonald, T. Kim, J. Werry, P. T. Simpson, F. Roosta, M. Trzaskowski, and Q. Nguyen. Stimage: robust, confident and interpretable models for predicting gene markers from cancer histopathological images. *bioRxiv*, pages 2023–05, 2023. Code: https://github.com/BiomedicalMachineLearning/STimage.

A. Vahadane, T. Peng, A. Sethi, S. Albarqouni, L. Wang, M. Baust, K. Steiger, A. M. Schlitter, I. Esposito, and N. Navab. Structure-preserving color normalization and sparse stain separation for histological images. *IEEE Transactions on Medical Imaging*, 35 (8):1962–1971, 2016.

S. Z. Wu, G. Al-Eryani, D. Roden, S. Junankar, K. Harvey, A. Andersson, A. Thennavan, C. Wang, J. Torpy, N. Bartonicek, T. Wang, et al. A single-cell and spatially resolved atlas of human breast cancers. *Nature genetics*, 53(9):1334–1347, Sept. 2021. ISSN 1061-4036. doi: 10.1038/s41588-021-00911-1. URL `https://www.ncbi.nlm.nih.gov/pmc/articles/PMC9044823/`.

R. Xie, K. Pang, S. Chung, C. Perciani, S. MacParland, B. Wang, and G. Bader. Spatially Resolved Gene Expression Prediction from Histology Images via Bi-modal Contrastive Learning. *Advances in Neural Information Processing Systems*, 36:70626–70637, Dec. 2023.

URL https://proceedings.neurips.cc/paper_files/paper/2023/hash/df656d6ed77b565e8dcdfbf568aead0a-Abstract-Conference.html.

Y. Zeng, Z. Wei, W. Yu, R. Yin, Y. Yuan, B. Li, Z. Tang, Y. Lu, and Y. Yang. Spatial transcriptomics prediction from histology jointly through transformer and graph neural networks. *Briefings in Bioinformatics*, 23(5):bbac297, 2022. Code: https://github.com/biomed-AI/Hist2ST.
